# Metabolomic profile and nucleoside composition of *Cordyceps nidus* sp. nov. (Cordycipitaceae): A new source of active compounds

**DOI:** 10.1371/journal.pone.0179428

**Published:** 2017-06-21

**Authors:** Juan Chiriví, Giovanna Danies, Rocio Sierra, Nicolas Schauer, Sandra Trenkamp, Silvia Restrepo, Tatiana Sanjuan

**Affiliations:** 1 Laboratory of Mycology and Plant Diseases, Universidad de los Andes, Bogotá, Colombia; 2 Product and Processes Design Group, Universidad de los Andes, Bogotá, Colombia; 3 Metabolomic Discoveries GmbH, Postdam-Golm, Germany; 4 Laboratorio de Taxonomía y Ecología de Hongos, Universidad de Antioquia, Medellín, Colombia; Universita degli Studi di Pisa, ITALY

## Abstract

*Cordyceps sensu lato* is a genus of arthropod-pathogenic fungi, which have been used traditionally as medicinal in Asia. Within the genus, *Ophiocordyceps sinensis* is the most coveted and expensive species in China. Nevertheless, harvesting wild specimens has become a challenge given that natural populations of the fungus are decreasing and because large-scale culture of it has not yet been achieved. The worldwide demand for products derived from cultivable fungal species with medicinal properties has increased recently. In this study, we propose a new species, *Cordyceps nidus*, which parasitizes underground nests of trapdoor spiders. This species is phylogenetically related to *Cordyceps militaris*, *Cordyceps pruinosa*, and a sibling species of *Cordyceps caloceroides*. It is found in tropical rainforests from Bolivia, Brazil, Colombia and Ecuador. We also investigated the medicinal potential of this fungus based on its biochemical properties when grown on four different culture media. The metabolic profile particularly that of nucleosides, in polar and non-polar extracts was determined by UPLC, and then correlated to their antimicrobial activity and total phenolic content. The metabolome showed a high and significant dependency on the substrate used for fungal growth. The mass intensities of nucleosides and derivative compounds were higher in natural culture media in comparison to artificial culture media. Among these compounds, cordycepin was the predominant, showing the potential use of this species as an alternative to *O*. *sinensis*. Furthermore, methanol fractions showed antimicrobial activity against gram-positive bacteria, and less than 3.00 mg of gallic acid equivalents per g of dried extract were obtained when assessing its total phenolic content by modified Folin-Ciocalteu method. The presence of polyphenols opens the possibility of further exploring the antioxidant capacity and the conditions that may enhance this characteristic. The metabolic composition and biochemical activity indicate potential use of *C*. *nidus* in pharmaceutical applications.

## Introduction

Consumer demand for natural products is growing worldwide. Fungi represent an important source of novel bioactive entities for drug development and therapeutic agents [1]. The ascomycete *Cordyceps* sensu lato (s.l.) is a large genus of arthropod-pathogenic fungi located in the order Hypocreales, some of which are traditionally used as medicinal mushrooms in China. Among these, the lepidopteran pathogen *Ophiocordyceps sinensis* (syn.*Cordyceps sinensis*) is perhaps the most widely renowned species with pharmaceutical properties [2]. This fungus is only found buried deep in the ground parasitizing larvae of the ghost moth *Thitarodes* spp (Lepidoptera: Hepialide) up to 5.000 meters of altitude in the eastern and southern parts of the Tibetan Plateau [3]. However, its supply is rapidly decreasing mainly due to its over-exploitation [4]. *Hirsutella sinensis* is currently recognized as its asexual state in cultivable species, based on the observation of its microcyclic conidiation and molecular analyses [5]. Unfortunately, its large-scale cultivation has not yet been achieved due to the fungus’ strong host dependence [6]. Thus, other species such as *Cordyceps militaris* and *Cordyceps pruinosa* which parasitize many genera of lepidopteran larvae have received further attention because they are generalist and have a similar metabolic composition as *O*. *sinensis* [4, 7–9].

The medicinal properties of *Cordyceps* s.l. have been mainly attributed to nucleosides, glycosylamines involved in the regulation and modulation of several biological processes and their derivatives [10–11]. These may be extracted from fruiting bodies or mycelia and are currently studied as therapeutic agents [7–8, 10, 12–13]. Nucleosides can be differentially induced by intrinsic or environmental factors, such as the developmental stage of the fungus, light exposure and wavelength, the composition of the culture media, among other factors [7–8], [12–15]. Traditionally, the nucleosides cordycepin (3’-deoxyadenosine), adenosine, inosine, guanosine, and uridine have been used as markers to assess the quality of natural and cultured species of *Cordyceps* [4, 16–17].

In this study, we propose a new species of *Cordyceps* that is pathogen of trapdoor spiders collected from tropical rainforest in the Neotropics. The metabolomic profile of this new taxon was also explored through the assessment of the intracellular extracts in mycelial growing on different culture media. The analysis focused in the nucleosides compounds and non-reported metabolites. Additionally, we evaluated the medicinal potential of this species by measuring antimicrobial activity and total phenolic content.

## Materials and methods

### Ethics statement

Collections were made in the first expedition under research permit number Res: 0871–2014 issued by *Corporación para el Desarrollo Sostenible del Sur de la Amazonía* (CORPOAMAZONIA) to Universidad de Antioquia. The second collection was made under research permit IDB0359 issued by *Ministerio de medio ambiente y desarrollo sostenible* to Universidad de los Andes.

### Field collections, culture, and maintenance

Six samples were collected in the Chicaque Natural Park, a cloud forest located in the Municipality of San Antonio del Tequendama, Cundinamarca, Colombia (elev. 1900 to 2700 m, average temperature of 14°C, and an average relative humidity of 85%). The second collection site, where three samples were collected, was the Amazonian tropical rainforest located in the Uitoto indigenous community from the Municipality of La Chorrera, Amazonas, Colombia (elev. 150 m, average temperature of 28°C, and an average relative humidity of 95%). The third location, where one sample was collected in June 2014, was El Amargal Biological Station Nuqui, a pacific tropical rainforest located in Chocó, Colombia (elev. 40 m, average temperature of 25°C, and an average relative humidity of 95%). The last collection site, where one sample was collected in November 2014, was in the municipal forest of Mariquita, a lowland tropical forest located in the inter Andes valley, Tolima, Colombia (elev. 560 m, average temperature of 28°C, and an average relative humidity of 75%). Four additional specimens were examined from the *Herbario Nacional del Ecuador* (QCNE), the *Herbario de la Universidad de Antioquia* (HUA) and Joao Araujo Personal collection.

The sampling process involved careful examination of the ground to observe the emergence of stromata from spider cadavers or nests. In the case of spider nests, we extracted the specimens by digging deep around the site to preserve the complete specimen. Subsequently, a small piece of tissue from a stroma was submerged and stored in cetyltrimethylammonium bromide (CTAB) buffer (1.4 M NaCl; 100 mM TriseHCl pH 8.0; 20 mM EDTA pH 8.0; 2% CTAB w/v) for posterior DNA extraction [18]. The remaining part of the specimen was placed in a single plastic bag with silica gel for transportation and posterior manipulation in the laboratory.

In sterile conditions, colonies were obtained by liberating ascospores from a piece (> 3 mm long) of stroma onto potato dextrose agar (PDA). These were then incubated at 25°C [19]. Sabouraud dextrose agar with 1% (w v^-1^) yeast extract (SDAY) medium was also used to maintain isolates. Strains were preserved on filter paper and conserved at 20°C [20].

Specimens and ex-type living cultures were deposited in the collection of the *Herbario de Antioquia* (HUA) and the fungal collection of *Museo de Historia Natural* of *Universidad de los Andes* (ANDES-F) in Bogotá, Colombia, where they are publicly available (S1 Table).

### Morphological studies

Collected material was observed under light microscopy after rehydrating in a 3% (w v^-1^) potassium hydroxide solution, and then staining with an aqueous solution of Congo red at 5% (w v^-1^) and lacto-fuchsine solution (0.1% (w v^-1^) of fuchsine acid dissolved in lactic acid). Width and length of perithecia, asci, ascospores, and partspores were measured using a MOBIC B3000 compound microscope. Methuen Handbook of Color was used for color descriptions of stromata [21].

Colony characteristics were described after growing strains on PDA and SDAY for 10 days at 25°C. A piece of mycelium was mounted into microscopic slides and stained with lactophenol cotton blue dye and lactofuchsine (0.1 g of acid fuchsine in 100 mL of lactic acid). Width of hyphae, width and length of phialides, and conidia were measured using a MOBIC B3000 and LEICA light microscope. A standard color chart was used to describe the color of colonies [22].

### DNA extraction, PCR, and sequencing

The small pieces of fresh tissue immersed in CTAB buffer were ground with sterile plastic pestles followed by the DNA extraction method described previously [23]. PCR was conducted to amplify of five nuclear loci, including the small and large subunits (SSU and LSU) of the nuclear ribosomal DNA (rDNA), the transcription elongation factor-1α (EF-1α), and the first and second largest subunits of the RNA polymerase II (RPB1 and RPB2). PCR reaction mixture and PCR program were carried out as previously described [24]. Forward and reverse nucleotide sequences were assembled and cleaned using Geneious 5.1.2v [25]. Sequences were manipulated in the software Bioedit Sequence Alingment editor software [26]. New rDNA sequences generated in this study are publicly available in GenBank (S1 Table).

### Phylogenetic analyses

In order to elucidate the taxonomic status of the fungal material from this study, we constructed a phylogenetic tree using the nuclear gene regions SSU, LSU, EF-1α, RPB2, and RPB1 from 212 taxa within the Hypocreales following previous studies [24,27–30]. A Maximum Likelihood analysis was first conducted using RAxML-VI-HPC v 2.0 as part of the CIPRES gateway [31]. Evolution model used was GTR-GAMMA with 1,000 bootstrap replicates [32]. The five nuclear loci were concatenated into a single dataset and eleven data partitions as were defined previously [23]. Subsequently, a Bayesian Inference analysis was conducted with MrBayes 3.2.6 and the evolutionary model and the partitions were specified as done for the RAxML analysis [33]. A total of 10,000,000 MCMCMC generations were performed using a sample frequency of 500 generations and a burn-in of 25% of the total run. Two runs using four chains each (one cold and three heated) were performed. Each run was examined with Tracer 1.5 to verify burn-in parameters and convergence of individual chains [34]. The phylogenetic trees generated in this study are publicly available in TreeBASE (ID: 20481). The final tree was edited in FigTree v1.4.0 (http://tree.bio.ed.ac.uk/software/figtree/) and Adobe Illustrator CS5 (Adobe Systems Inc., CA, USA).

### Assessment of the metabolic profile

To explore the metabolic composition of the trapdoor spider pathogen, the most recently collected strain, ANDES-F 1080, was grown on four distinct culture media: *i*) SDAY as a common and standard artificial culture medium, *ii*) SDAY with 5 ppm of sodium selenite (Sigma, United States) (SDAY-SS) as suggested for the enhancement of nucleoside production [16], brown rice (BR) medium as a semi-natural culture medium used for production of biomass and of reproductive structures in *Cordyceps* [35], and *iv*) an integument medium made from tarantulas (TA) to examine components produced on a substrate similar to that of the host. A conidial suspension was prepared and adjusted to 105 spores per mL in sterile distilled water with 0.1% (v v-1) Tween 80. Inoculum was prepared by mixing 1 mL of the conidial suspension with an equal volume of Sabouraud Dextrose broth with 1% (w v-1) of yeast extract (SDY), and then incubated at 25°C for 3 days. Subsequently, the entire suspension was added into a 250 mL glass beaker containing 50 mL of each of the four culture media tested. Each medium with the conidial suspension added, was incubated at 25°C for one month with a photoperiod of 12:12. Colony color and medium color were described as deep orange (N00A99M80), orange (N00A80M80), or pale orange (N00A50M50), based on the color chart previously used for cultures. Mycelial density from four-weeks-old fungal cultures was classified as thin (+), moderate (++), or compact (+++).

All culture media were prepared with 1.5% (w v-1) agar to avoid bias by the presence or absence of this component. The proportion of brown rice and water used for the BR medium was 50:60 w v-1. The tarantula integument medium was prepared from adult Theraphosidae spiders, which were deeply sedated in an ethyl acetate chamber prior to the euthanization as is suggested by the Institutional Animal Care and Use Committee [36]. Dead specimens were frozen at -20°C, lyophilized, ground, and finally added into the medium in a proportion of 20 g L-1.

### Metabolite extraction method

Destructive sampling was used weekly and consisted of a hard separation of all fungal biomass from each of the culture media. This biomass was kept at -20°C until it was lyophilized and ground for posterior extraction of metabolites. Dry-weight from four-weeks-old fungal cultures was assessed. Three fractions of extracts deriving from three solvents: n-hexane as a non-polar solvent and methanol and water as polar solvents were included. These fractions were used for metabolomic profiling and antimicrobial assays. Methanol fractions from one, two, and three-weeks-old cultures were used to assess antioxidant properties through time.

Methanol and n-hexane extractions were performed as it was described for *C*. *pruinosa* with some modifications [8]. Briefly, the extraction consisted on macerating 100 mg of the fungal material and adding 2 mL of solvent. Once an emulsion was obtained, samples originating from methanol and n-hexane fractions were sonicated and then centrifuged at 2,000 rpm for 10 min. The supernatant from these samples was carefully recovered and finally filtered through a membrane of 0.22 μm. In the case of the water-based extraction, samples were treated with the ambient temperature water extraction protocol to optimize the extraction of nucleosides [16].

### Metabolomic screen and statistical analyses

Polar and non-polar fractions were lyophilized and dried, respectively, and then adjusted to a final concentration of 4 mg of extracted fungal biomass per 1 mL of solvent. Five replicates of these diluted solutions from 12 combined treatments (each culture medium used for fungal growth and each solvent used for extraction) were analyzed by gas and liquid chromatography (GC and LC, respectively) coupled to mass spectrometry. All subsequent steps were carried out at Metabolomic Discoveries GmbH (Potsdam, Germany; http://www.metabolomicdiscoveries.com/). Derivatization and analyses of metabolites was performed by a GC-MS 7890A (Agilent, Santa Clara, USA) [37].]. The LC separation was performed using hydrophilic interaction chromatography with a ZIC-HILIC 3.5 μm, 200 A column (Merck Sequant, Umeå Sweden), operated by an Agilent 1290 UPLC system (Agilent, Santa Clara, USA). Acetonitrile was used as the LC mobile phase consisting of a first linear gradient ranging from 90% to 70% over 15 min, followed by a linear gradient ranging from 70% to 10% over 1 min, a 3 min wash with 10%, and a final re-equilibration wash with 90% for 3 min. The flow rate was 400 μL min^-1^ with 1 μL of injection volume. The mass spectrometry was performed using a high-resolution 6540 QTOF/MS Detector (Agilent, Santa Clara, USA). The measured metabolite concentration was normalized to internal standard concentrations. Metabolites were identified by using Metabolomic Discoveries' database entries of authentic standards.

The concentration of metabolites in the different treatments was tested for normality (Shapiro-Wilk-Test) and variance homogeneity (F-Test) using the appropriate statistical tests (Students test, Welch test, and Mann-Whitney test). Changes in the concentration of metabolites among treatments were considered significant with a p-value < 0.05. Venn diagrams and principal component analyses (PCAs) were used to visualize distinct segregations between metabolites from the 12 groups studied. Volcano graphs were performed to confirm significant differences between metabolites from different samples. Statistical analyses were done using the software JMP Genomics 5.1 (SAS Institute Inc., Cary, NC, 1989–2007). Nucleoside composition was depicted in pie charts and bar diagrams. An ANOVA test was performed to confirm differences among mass intensities.

### Antimicrobial susceptibility tests

Reference bacterial and fungal strains were obtained from the American Type Culture Collection and National Collection of Type Cultures. To assess the antimicrobial properties of the metabolites produced by the fungal strain ANDES-F 1080, nine gram-positive bacteria (*Staphylococcus aureus*, *Staphylococcus haemolyticus*, *Staphylococcus epidermidis*, *Kocuria rosea*, *Enterococcus faecalis*, *Enterococcus faecium*, *Bacillus cereus*, *Bacillus circulans*, and *Lysinibacillus sphaericus*), five gram-negative bacteria (*Enterobacter aerogenes*, *Salmonella typhimurium*, *Salmonella paratyphi B*, *Escherichia coli*, and *Pseudomonas aeruginosa*), three molds (*Fusarium oxysporum*, *Fusarium solani*, and *Aspergillus fumigatus*), and three yeasts (*Candida albicans*, *Candida parasilopsis*, and *Candida kruseii*) (S2 Table) were included in our study. All strains were kept at 4°C and grown as described below prior to the susceptibility assays. Bacterial strains were grown in nutrient agar medium at 37°C for 24 h, while fungal strains were grown in SDAY medium at 25°C for 5 days.

Bacterial and yeast inoculations were adjusted to a 0.5 McFarland turbidity (1–2 x 106 CFU mL^-1^) and spread evenly over the entire surface of the medium using a sterile cotton swab. Inocula for molds were prepared as described before [38–39]. The susceptibility of the microorganisms was determined using a well diffusion assay on Mueller-Hinton (MH) agar (Scharlau, Barcelona, España), following standard procedures as described previously [40]. Extracts originating from four-weeks-old fungal cultures were tested by adding 20 μL of the extract in each individual well. Methanol fractions were lyophilized and then resuspended on water to avoid bias by the toxicity of this solvent. Wells with gentamicin (Sigma-Aldrich, St. Louis, MO, USA), amphotericin (Sigma-Aldrich, St. Louis, MO, USA), and chlorhexidine (Sigma-Aldrich, St. Louis, MO, USA) at 100 μg mL-1 were used as positive controls for bacteria, molds, and yeasts, respectively. Wells containing water and n-hexane were used as negative controls. Microorganisms were incubated under standard conditions as described above. All experiments were carried out in triplicate. Mean and standard deviation were calculated and used as the central tendency unit and the statistical dispersion unit, respectively. Zones of growth inhibition were examined and the diameter of each zone was measured and recorded. Fractions that showed antimicrobial activity were later tested to determine the Minimal Inhibitory Concentration (MIC) for each microorganism. The MIC assay was done in triplicate starting with a concentration of 50 mg of fungal material per 1 mL of solvent and diluting by half the concentration until an inhibition halo was no longer observed.

### Determination of total phenolic content

The total phenolic content (TPC) of methanol fractions was measured using the modified version of the Folin-Ciocalteu method [8]. The results were estimated using a calibration curve of gallic acid (Sigma-Aldrich, St. Louis, MO, USA) at a concentration ranging from 100 to 500 ppm; results are expressed as mg of gallic acid equivalents (GAE) per g of dried extract. A total of eight replicates of each TPC assessment were conducted. Mean and standard deviation were calculated and used as the central tendency unit and the statistical dispersion unit, respectively. Differences between data obtained from the same week were compared by conducting a Student’s t-test through the statistical software R v 3.0.1[41]. A p-value < 0.05 was considered as significant.

### Nomenclature

The electronic version of this article in Portable Document Format (PDF) in a work with an ISSN or ISBN will represent a published work according to the International Code of Nomenclature for algae, fungi, and plants, and hence the new names contained in the electronic publication of a PLOS ONE article are effectively published under that Code from the electronic edition alone, so there is no longer any need to provide printed copies.

In addition, new names contained in this work have been submitted to MycoBank from where they will be made available to the Global Names Index. The unique MycoBank number can be resolved and the associated information viewed through any standard web browser by appending the MycoBank number contained in this publication to the prefix http://www.mycobank.org/MB/. The online version of this work is archived and available from the following digital repositories: PubMed Central (www.ncbi.nlm.nih.gov/pubmed) and LOCKSS (www.lockss.org/).

## Results

### A new fungal pathogen of trapdoor spiders

In this study, 15 specimens were examined from parasitized large spiders belonging to the suborder Mygalomorpha. Four specimens were found associated with tarantula spiders (Theraphosidae) and presented a morphology that resembles the concept of Petch from 1933 of *Ophiocordyceps caloceroides* [42]. These specimens showed threadlike stromata that were 10–40 cm long, a fertile area distinguished by its pruinosa appearance due to the large ostioles on the tiny perithecia (90–250 μm), and short sub-fusiform asci with slightly curved fusiform multiseptate ascospores. In contrast, the other eleven specimens that were associated to trapdoor spiders (Idiopidae and Barynchelidae), had slightly capitate stromata that were less than 4 cm long and a fertile area that was cylindrical to clavate. They also had large perithecia (300–500 μm) with cylindrical asci and filiform ascospores breaking into irregular partspores.

**Fig 1:**
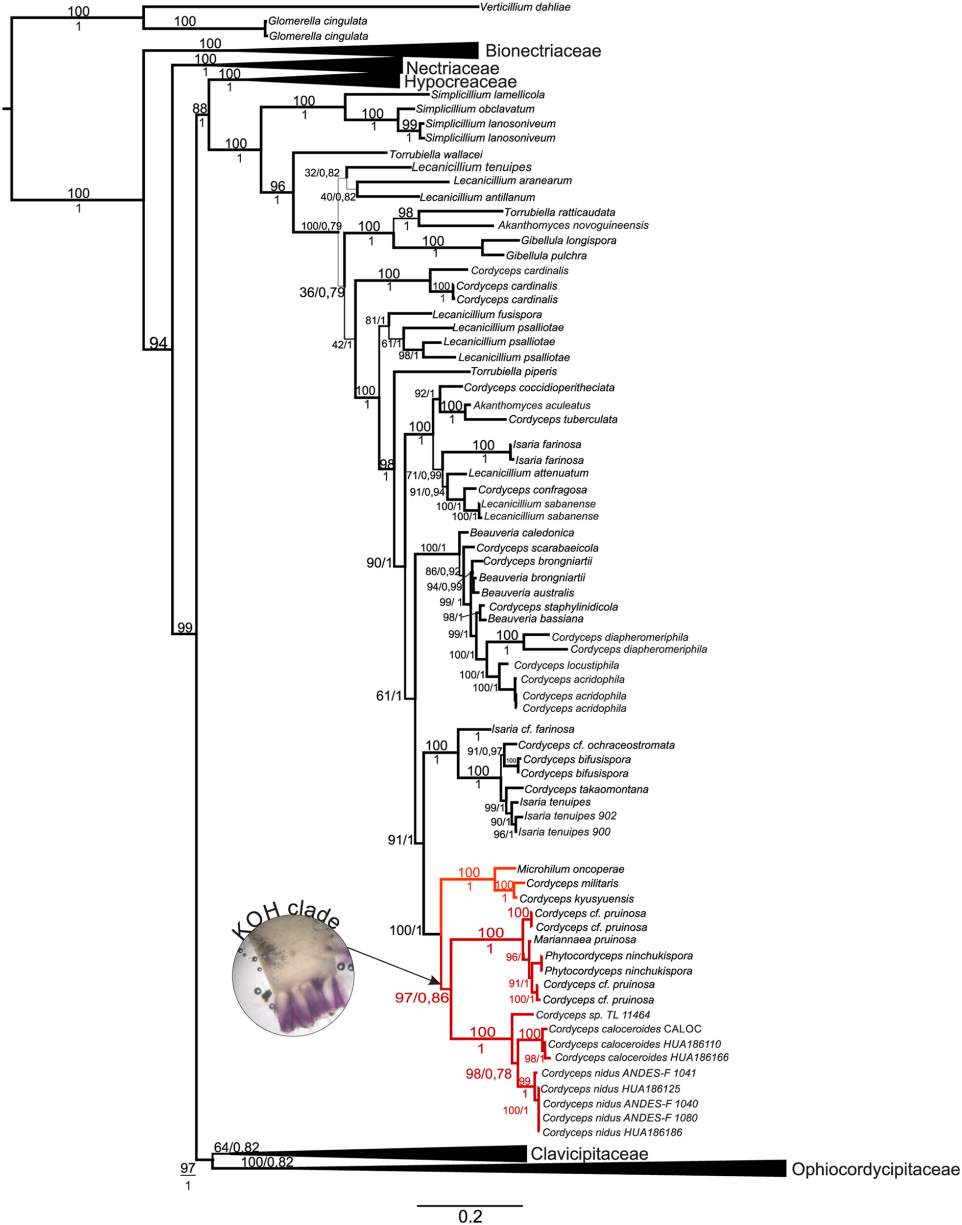
Phylogenetic relationships inferred from a Maximum Likelihood analysis of *Cordyceps* species which parasitize spiders. Combined data set of SSU, LSU, TEF, RPB1, and RPB2 nuclear loci. Sequences from *Verticillium dahlie* and *Glomerella cingulata* were used as outgroups. Numbers above branches indicate Bootstrap support (BS) and below posterior probability support in Bayesian analyses (PP). Bionectriaceae, Nectriaceae, Hypocreaceae, Clavicipitaceae, Ophiocordycipitaceae are collapsed to emphasize of Cordycipitaceae.

We merged the thirty-three sequences obtained from these specimens with a data set comprising 227 taxa from six families (Bionectriaceae, Nectriaceae, Hypocreaceae, Cordycipitaceae, Clavicipitaceae, and Ophiocordycipitaceae) into a unique concatenated alignment of five loci comprising 4498 base pairs (1115 bp SSU, 977 bp LSU, 1050 bp EF-1α, 1065 bp RPB2, and 791 bp RPB1). Our samples grouped into a new taxon with a strong statistical support (bootstrap, MLBS = 99; PP = 1) inside the Cordycipitaceae family (Fig 1). They were located in the same clade as *Cordyceps pruinosa* and *Phytocordyceps ninchukispora*, with the specimens of *Ophiocordyceps caloceroides* placed as sibling species. Additionally, *Cordyceps* sp. TL11464 was placed close to *O*. *caloceroides* sharing the same ancestor.

We proposed *Cordyceps nidus* sp. nov. as a new species of fungal pathogen of trapdoor spiders (Mygalomorpha: Idiopidae). It is a sibling species of *Cordyceps caloceroides*, a pathogen of tarantula spiders (Mygalomorpha: Theraphosidae), which has been classified previously as *O*. *caloceroides* base on asci morphology [30]. Both species, together with *C*. *pruinosa*, form a clade here referred to as KOH+ (Fig 1) due to the shift in color from the typical red pigmentation to violet when a drop of potassium hydroxide is added.

### Taxonomy

**Fig 2:**
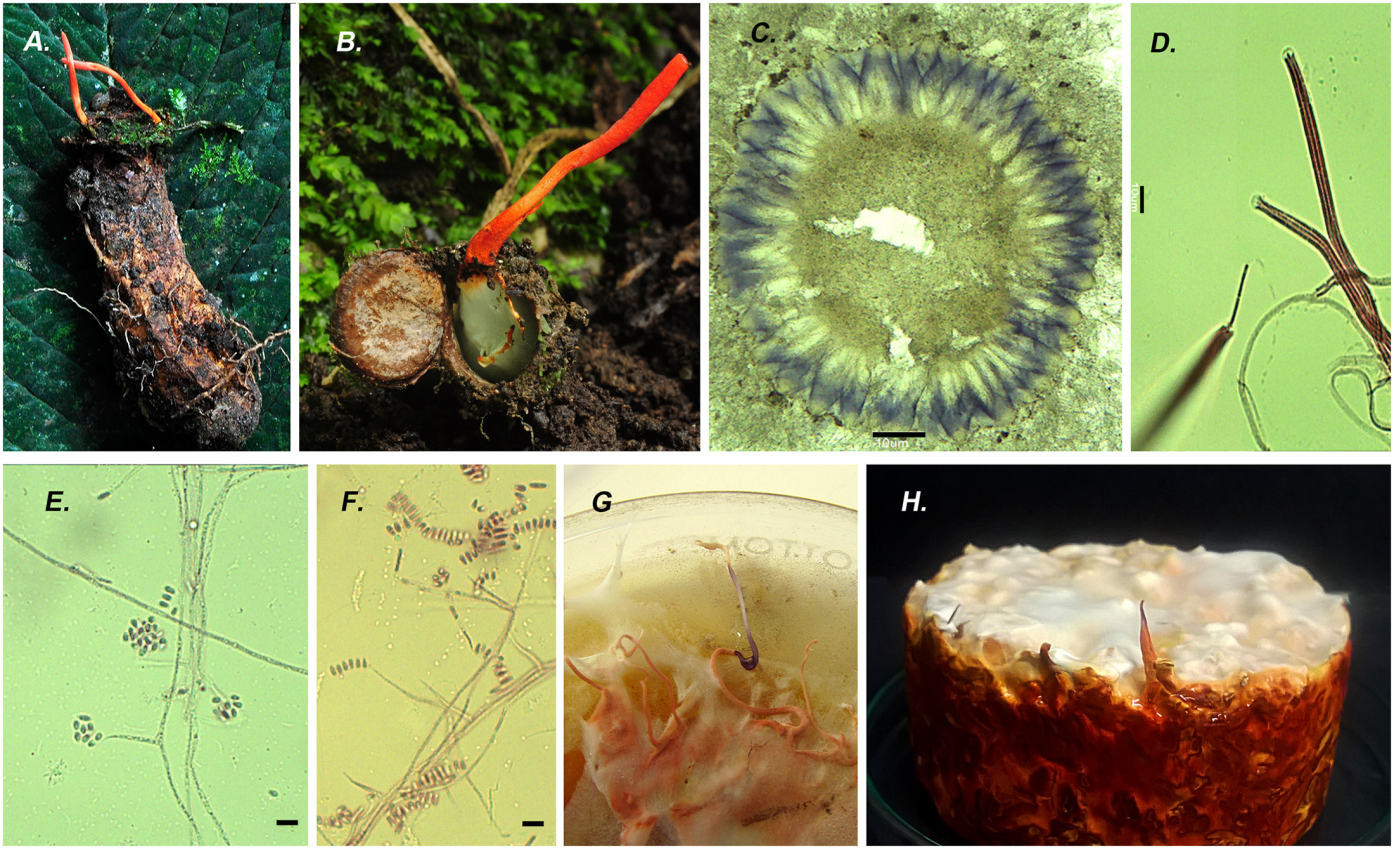
*Cordyceps nidus*. **A)** Stromata emerging from a nest of trapdoor spider from the family Idiopidae ANDES-F 1080 **B)** Inside of the nest with the red stromata on the lid HUA 186125. **C)** Cross-section of perithecia turned purple with KOH. **D)** Asci and ascospores stained with fucsin acid dye. **E-G)** Conidial state of *C*. *nidus* E) Long and slender phialides with a conidial head produces on prostate narrow hypha. **F)** Conidia in a row showing different shapes and sizes. **G)** Synnemata isolated in PDA turned purple on the tip due to the reaction with KOH 3%. **H)** Synnemata Isolated on brown rice showed strong red pigments after 30 days. Scale bars: C = 100 μm 10 ×, D-F = 10 μm 100 ×.

***Cordyceps nidus*** T. Sanjuan, J.S. Chiriví-Salomón & S. Restrepo sp. nov. Fig 2.

[*urn*:*lsid*:*mycobank*.*org*:*names*: *MB817601*]

*Etymology*: In reference to the burrow of the trapdoor spiders.

*Description*: Stromata claviform, simple, gregarious, fleshy, 10–42 mm long. Fertile part subcylindrical, apex rounded, pruinosa, coral-red (8B6) to carmine red (9A8) 2.5−18 × 0.5–3 mm. Stipe terete, smooth, brownish red (9C7), 5−34 × 0.5−2 mm. Perithecia pseudoimmmersed, perpendicular orientation, ellipsoid, 300–500 (-630) × 110–190 (-205) μm (n = 40). Asci cylindrical, (145-) 190–360 × 2–4 μm (n = 50), cap 3–4.2 × 1.2–3 μm. Ascospores filiform, hyaline, 100–120 × 1.0 μm (n = 5); breaking irregularly into truncate partspores, (4-) 6–10 × 1μm (n = 50).

*Conidial state*: Colonies on PDA slow-growing, reaching 28.5–30.0 mm in diameter in 10 days at 25°C, white (N00A00M00) to pale orange (N00A50M50), velvety; reverse deep orange (N00A99M80) to pale orange (N00A50M50), margin entire, soluble pigments not produced. Colonies on SDAY slow-growing, reaching 20.0−35.0 mm in diameter in 10 days at 25°C, pale orange (N00A50M50), tomentose; reverse deep orange (N00A99M80), margin entire, red pigments produced on culture media. Fungal odor. Vegetative hyphae 1.0−2.0 μm wide, smooth-walled, regularly septate. Conidiophores arising from submerged hyphae, moderately branched. Phialides solitary, 5.0−70.0 × 1.0−2.0 μm (n = 30). Conidia forming linear and globose mucilaginous heads, ellipsoidal to ovoid, usually straight, smooth-walled, 1-celled, 2.0−7.0 × 1.0−2.0 μm (n = 30).

*Host*: Stromata emerging from the lid of the burrow of young trapdoor spiders belong to Idiopidae family (Aranae: Mygalomorpha). White mycelia cover the spider and the burrow like a net connected to the stromata on the lid.

*Specimens examined*: COLOMBIA, Cundinamarca, San Antonio del Tequendama, Chicaque Natural Park, GPS: 4°36'28.51″N, 74°18'27.50''W, elev. 2,500 m, on the burrow of Idiopidae spiders. 27 Feb 2011, T. Sanjuan 903, Holotype: HUA 186125.

*Additional specimens examined*: COLOMBIA, Cundinamarca, San Antonio del Tequendama, Chicaque Natural Park, GPS: 4°36'28.51''N, 74°18'27.50''W, elev. 2,500 m. 27 Feb 2011, T. Sanjuan 904, Isotype: HUA 186186, 10 de Apr de 2011, T sanjuan 930, 12 Nov 2013, T. Sanjuan 1129, 17 Feb 2015, T. Sanjuan 1163, Paratype: ANDES-F 1080. Amazonas, La Chorrera, San Francisco Uitoto community, GPS: 1°26'58''S, 72°47'38''W, elev. 150 m, 11 Sep 2011, T. Sanjuan 989 Paratype: ANDES-F 1040. Puerto Santander, Araracuara Canyon, GPS: 0°37'21''S 72°24'26''W, alt 115 m. 15 May 2009, T. Sanjuan 728, HUA 186141. Chocó, Nuquí, Biological Station El Amargal, GPS: 5°34'39''N77°30'49'' S, elev. 40 m, 29 Jun 2014, T. Sanjuan 1141, ANDES-F 1029. Tolima, Mariquita Municipal Forest, 5°11'29''N 74°54'40''W, elev. 560 m, 11 Nov 2014, T. Sanjuan 1161, ANDES-F 1246. ECUADOR, Pichincha, Canton Quito, protected forest Guajalito river, 00°15'54''S 78°40'00''W, 2400 m, 20 Dic 2003 I. Vargas 022, QCNE 186229.

*Living cultures*: COLOMBIA, Cundinamarca, San Antonio del Tequendama, Chicaque Natural Park, GPS: 4°36'28.51''N, 74°18'27.50″O, elev. 2.500 m. 17 Feb 2015 T. Sanjuan 1163 Paratype: ANDES-F 1080, 3 Dic 2015, T. Sanjuan 1202, ANDES-F 1247. Tolima, Mariquita Municipal Forest, GPS: 5°11′29″N 74°54′40″W, 560 m, 11 Nov 2014, T. Sanjuan 1161, Isotype: ANDES-F 1248.

*Known distribution*: Tropical rain forest of Colombia and Ecuador.

***Cordyceps caloceroides*** Berk. & M.A. Curtis, J. Linn. Soc. 10: 375. 1868 Fig 3

**Fig 3:**
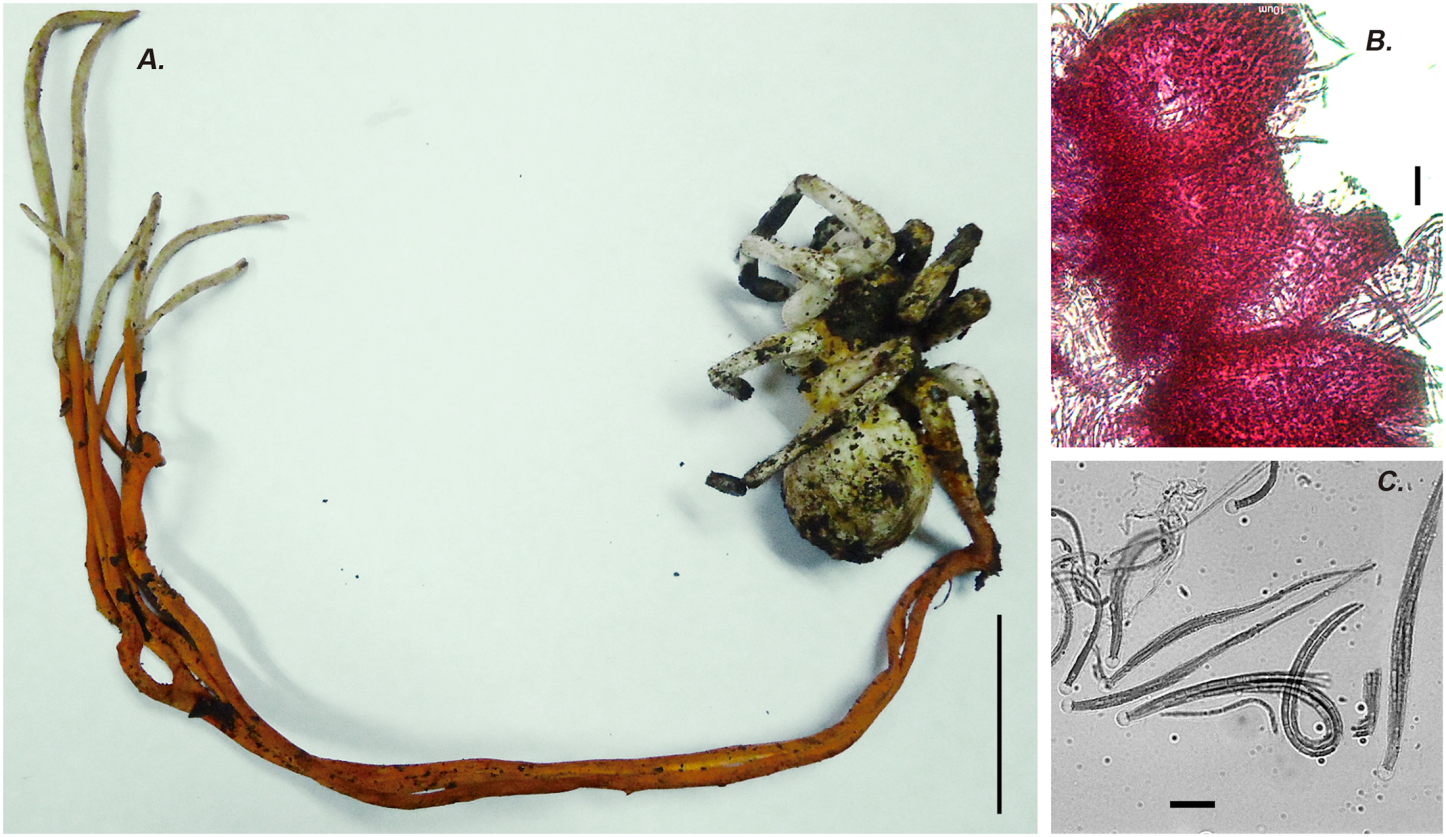
*Cordyceps caloceroides*. **A)** A long stromata emerging from a big Theraphosidae spider (picture taken by Carlos Padilla on Yasuni Scientific Research Station, EC). **B)** Cross section of perithecia stained with Fucsin lactic acid showing the broad ostioles HUA 186166. **C)** Asci stained with cotton blue dye showing the curved filiform ascospores Scale bars: A = 5 cm, B = 10 μm 40 ×, C = 10 μm 100 ×.

[*urn*:*lsid*:*mycobank*.*org*:*names*: *MB8184173*]

= *Ophiocordyceps caloceroides* (Berk. & M.A. Curtis) Petch Trans. Brit. Mycol. Soc. 18: 63. 1933.
= *Cordyceps wittii* Henn. Bot. Jahrb. Syst. 23: 539. 1897.

*Specimens examined*: COLOMBIA, Amazonas, La Chorrera, San Francisco Uitoto community, GPS: 1°26'58''S, 72°47'38''W, elev. 150 m, 22 May 2010, T. Sanjuan 871, HUA 186166. Tarapaca, El Zafire Reserve, 4°0'21'' S, 69°53'55'' W, elev. 150 m. 6 Jan 2008, A. Vasco 1962, HUA 186110. ECUADOR, Orellana, Yasuni National Park. Tiputini Research Station, GPS: 0°38'14.84''S 76°09'1.83''W, elev. 200 m, 13 Jul 2004, T. Sanjuan 460, QCNE186272; Chimborazo, Canton Penipe, Sector Palictahua, 01°31'S, 78°29' W, elev. 2,400 m, Nov 1 2003, N. Erazo 003, QCNE 186235. BRAZIL, Amazonas, Reserva Adolpho Ducke, 02°57'42''S, 59°55'40''W, elev. 100 m. JPM Araujo CALOC.

*Host*: Stromata emerging from the joins on the body of Tarantula spider belong to Theraphosidae family (Aranae: Mygalomorpha), located inside of its burrow (Fig 3A)

*Note*: One additional specimen from Ecuadorian cloud forest which fit the morphology of *C*. *caloceroides* sensu stricto was examined. It was collected in April 2002 by Bryce Kendrick. It is part of the exhibition of the Vancouver Mycological Society (http://mushroomobserver.org/139860?q=1Xxc).

*Known distribution*: tropical rainforest in Bolivia, Brazil, Colombia, Cuba and Ecuador.

### *Cordyceps nidus* produced greater biomass on brown rice culture medium

In general, *C*. *nidus* showed the following characteristics: orange (N00A80M80) to white (N00A00M00) colonies, with a deeper pigmentation on the reverse, a characteristic fungal smell, and a typical tomentose texture. In spite of the low pigmentation on BR, *C*. *nidus* showed higher values of dry-weight and mycelial density on this medium compared to the others. The pigmentation on all cultures intensify through time, but pigments on BR intensify less and were pale orange (N00A50M50) almost all the time. Nevertheless, orange (N00A80M80) pigments were observed on the reverse of the colony that diffused into the BR medium. Fungal growth on SDAY and SDAY-SS was similar on all measures recorded. The lowest values of dry weight for C. nidus were obtained on isolates grown on TA culture medium (Table 1).

**Table 1:** Effect of culture medium on mycelial growth of *C*. *nidus* after four weeks.

Culture medium	Dry-weight	Mycelial density	Pigmentation	Code color [22]
SDAY	1225.32 (± 65.13)	++	Deep orange	N00A99M80
SDAY-SS	1198.43 (± 87.23)	++	Deep orange	N00A99M80
BR	1875.43 (± 71.68)	+++	Pale orange	N00A50M50
TA	802.41 (± 98.45)	+	Orange	N00A80M80

SDAY, Sabouraud Dextrose Agar supplemented with yeast extract; SDAY-SS, SDAY supplemented with sodium selenite; BR, brown rice agar; TA, tarantula integument agar.

### The metabolite profile is highly dependent on the medium used to grow the fungus

The metabolic profile of extracts of *C*. *nidus* grown on four distinct culture media showed a high diversity of compounds (S3 Table). Water fractions obtained from all culture media treatments resulted in a total of 3744 compounds, from which 17.17% of metabolites were non-dependent on the substrate. We also found that BR showed the largest number of unique compounds (5.07%), followed by TA (2.30%), then SDAY-SS (1.76%), and finally SDAY (0.03%). On the other hand, methanol fractions summed a total of 3,271 compounds, where 41.18% were shared among all culture media treatments. In this case, TA produced the greater number of unique compounds (5.53%), followed by SDAY (3.73%), BR (3.33%), and SDAY-SS (2.48%). Finally, n-hexane fractions showed a low number of extracted metabolites with a total of 1,588 compounds. From these extracts, 31.36% of all metabolites were shared among all treatments. Similar quantities of unique compounds were found in each culture media treatment when using n-hexane as solvent (S1 Fig).

**Fig 4:**
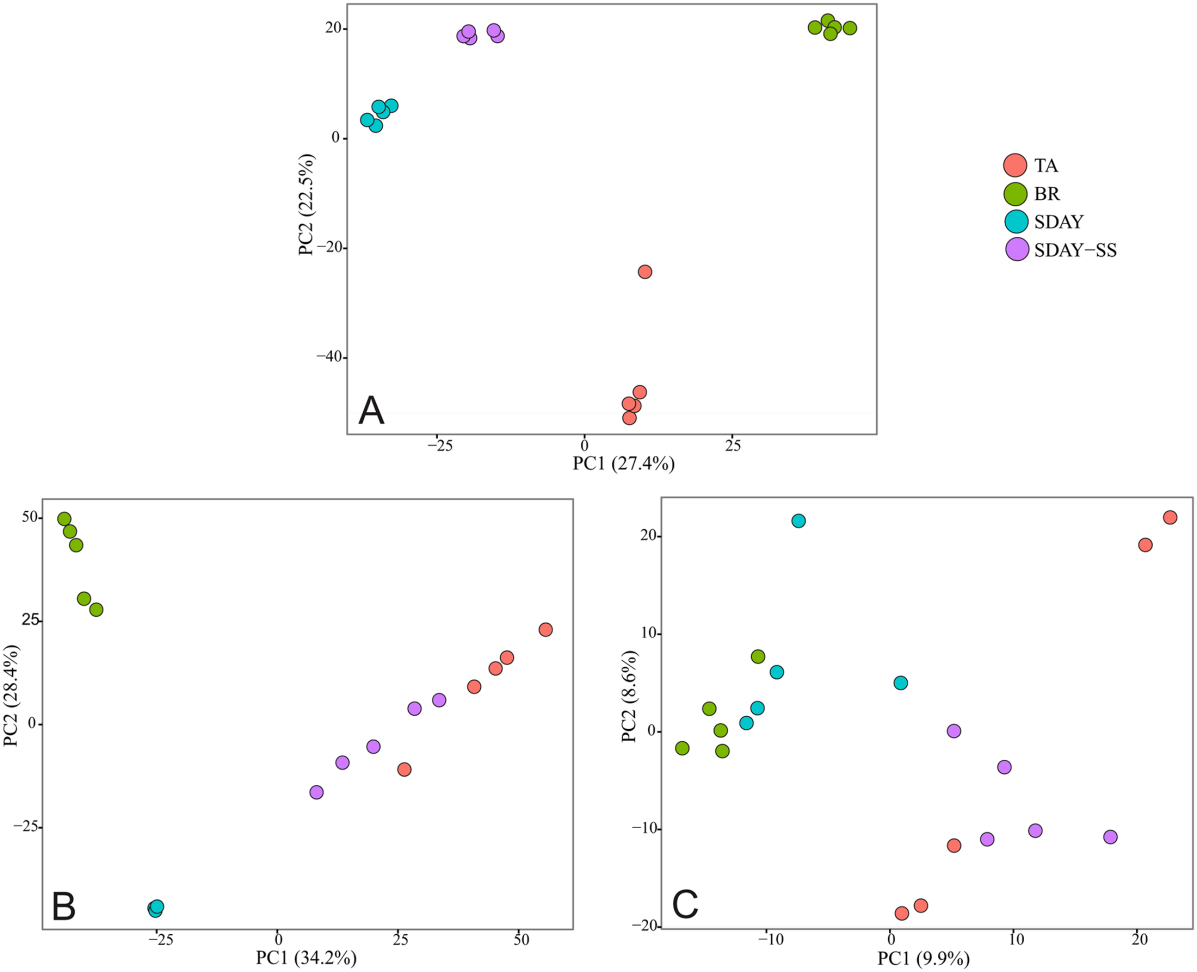
PCA analysis for the culture medium across different solvent fractions. A) methanol, B) water, and C) n-hexane extractions.

In addition, the mass intensity of each metabolite was highly distinct. This is directly related to the concentration of metabolites in each of the extracts. The proportion of metabolites, especially those that were extracted with methanol, was found to be highly dependent on the culture medium used to grow *C*. *nidus* (Fig 4). Water and n-hexane fractions presented a strong matrix effect, which is related to high concentration of metabolites, this leads to a lower detection rate of them. For methanol fractions, the first and second principal components (PC 1 and PC 2) explained 27.45% and 22.5% of the variation, respectively. In the water fractions, PC 1 and PC 2 explained 34.2% and 28.4% of the variation, respectively. For the n-hexane fractions, no clear differences were observed among culture media, and the combined PCs only explained 18.5% of the variation.

### Semi-natural culture media induced the highest production of nucleosides

**Fig 5:**
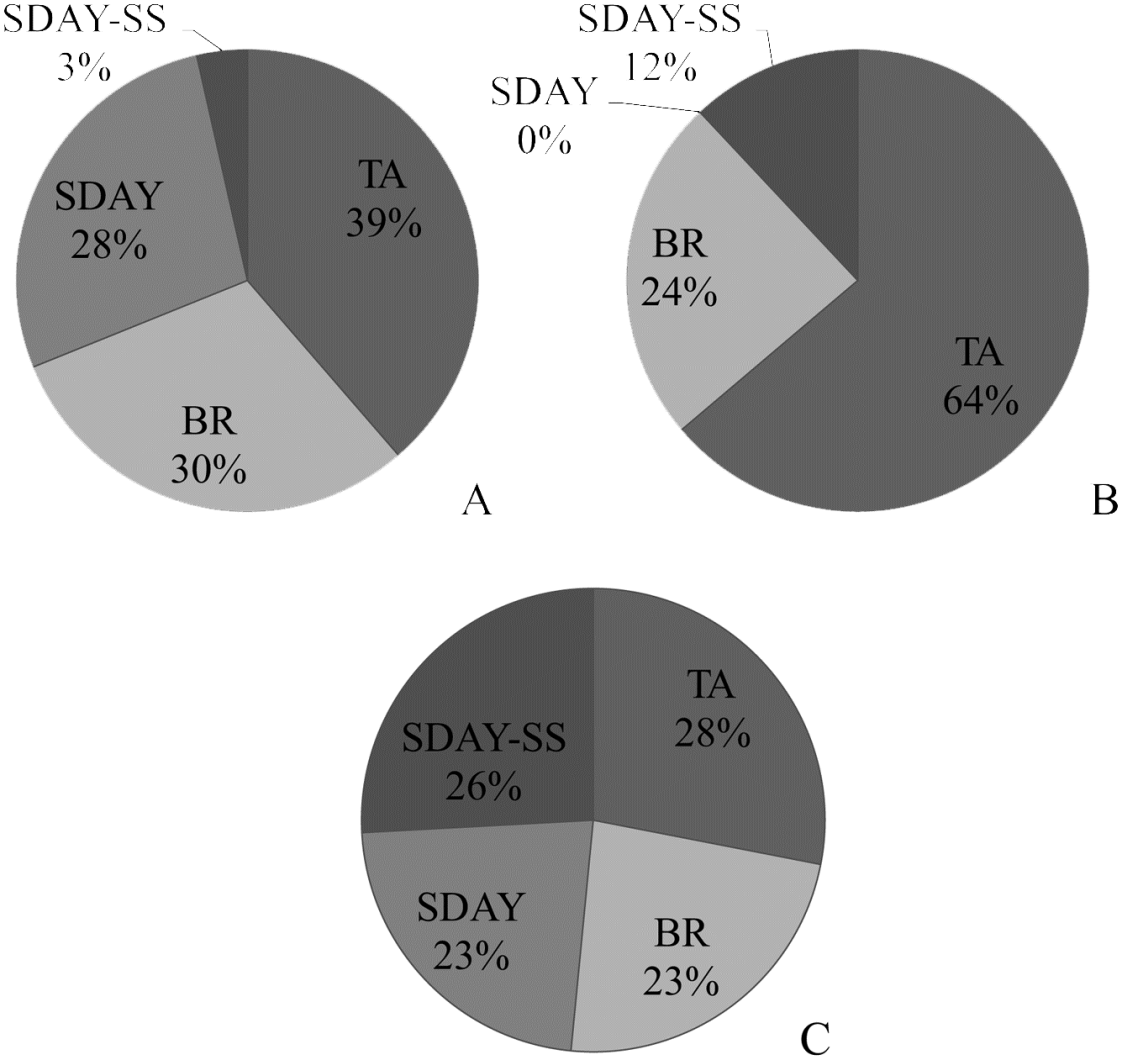
Nucleoside composition of from *C*. *nidus* grown on different culture media. **A)** methanol, **B)** water, and **C)** n-hexane extracts.

**Fig 6:**
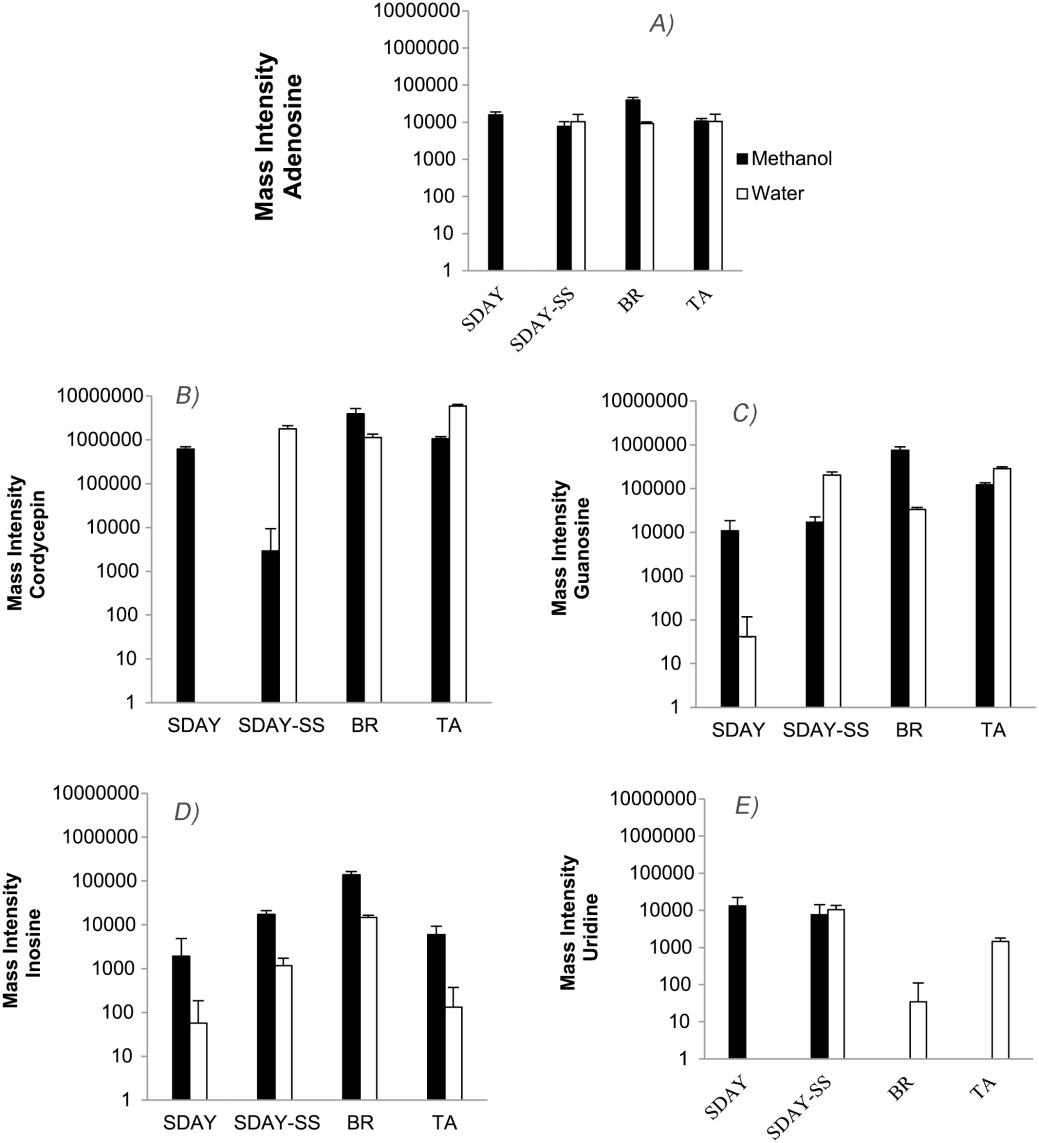
Mass intensities of nucleosides. **A)** cordycepin, **B)** adenosine, **C)** inosine, **D)** guanosine, and **E)** uridine extracted from mycelium of *C*. *nidus* grown on different culture media. Methanol (black) and water (white) extracts. Statistical significance for alpha = 0.05: a. 9.26 10^−9^, b. 1.32 10^−7^, c. 5.83 10^−11^, d. 4.11 10^−11^, e. 3.72 10^−3^, f. 2.85 10^−3^, g. 1.64 10^−14^, h. 2.39 10^−12^, i. 1.44 10^−14^.

The content of nucleosides in *C*. *nidus* was compared among the different culture media and extraction solvents used (Table 2, Fig 5). Certainly, when polar solvents were used for extraction, a richer set of nucleosides was observed due to their chemical affinity for polar solvents. Mass intensity values of nucleosides were detected higher in semi-natural culture media (BR and TA) than in the artificial media tested (SDAY and SDAY-SS). In contrast, the content of nucleosides of the non-polar fractions was similar among the different culture media used for fungal growth (Fig 5). Among the nucleosides detected, abacavir, cordycepin, and N6-hydroxyethyl adenosine predominated with mass intensity values above 10^6^ (Table 2). In water fractions, the mass intensity value of 5’-Methylthioadenosine also reached this order of magnitude. Mass intensity values of nucleosides used as markers of quality control of *Cordyceps* products were thoroughly studied in polar solvents (Fig 6). Data set from n-hexane fractions was excluded from this analysis due to its strong matrix effect and the undifferentiated nucleoside composition (Figs 4 and 5). Remarkably, the mass intensities reported for cordycepin were the highest reaching values around of 10^6^ (Fig 6B), while the other markers were reported with mass intensity values around of 10^4^ (Fig 6A and 6C–6E).

**Table 2:** Nucleosides and derivatives obtained from *C*. *nidus* grown on the different culture media. The entries in the table are the means and standard deviations of the mass intensities.

METABOLITES	SDAY	SDAY-SS	BR	TA
***Methanol fractions***				
(S)(+)-Allantoin	2.06 10^4^ ± 1.35 10^4^	3.60 10^3^ ± 8.052 10^3^	-	-
(S)-5'-Deoxy-5'-(methylsulfinyl)adenosine	1.89 10^5^ ± 1.80 10^4^	1.59 10^5^ ± 2.69 10^4^	1.86 10^3^ ± 1.23 10^3^	8.66 10^4^ ± 9.82 10^3^
1-Methylguanosine*	0.19 ± 0.38	-	21.36 ± 11.47	0.31 ± 0.47
1-Methylinosine	-	1.26 10^4^ ± 7.70 10^3^	-	1.86 10^3^ ± 4.17 10^3^
2-Aminoadenosine*	2.95 ± 2.17	1.86 ± 1.45	10.81 ± 9.40	-
2-Methylaminoadenosine	1.42 10^4^ ± 1.32 10^4^	9.00 10^3^ ± 3.55 10^3^	6.40 10^3^ ± 1.68 10^3^	4.98 10^4^ ± 9.51 10^3^
3’-amino-3’-deoxyadenosine*	17.17 ± 11.76	22.71 ± 11.88	22.86 ± 13.51	14.74 ± 4.12
3’-homocitrullyl-amino-3’deoxyadenosine	2.33 10^3^ ± 3.29 10^3^	-	-	3.18 10^4^ ± 827.76
3-Methyluridine	2.65 10^4^ ± 3.63 10^3^	1.97 10^4^ ± 4.05 10^3^	8.60 10^3^ ± 5.05 10^3^	4.38 10^3^ ± 5.99 10^3^
5’-(aminoiminomethyl)amino-5’-deoxyadenosine dihydrogen sulfate*	1.25 ± 1.47	-	1.04 ± 0.68	0.66 ± 1.02
5-Carboxy-2’-deoxyuridine	-	-	8.68 103 ± 1.23 10^3^	-
5’-Dehydroadenosine*	1.11 ± 0.38	0.24 ± 0.45	10.01 ± 3.09	8.30 ± 1.83
5-Methyldeoxycytidine	3.76 10^4^ ± 1.06 10^4^	1.88 10^4^ ± 6.20 10^3^	123.31 ± 275.73	6.71 10^4^ ± 2.62 10^4^
5'-Methylthioadenosine	5.40 10^5^ ± 6.82 10^4^	2.21 10^5^ ± 6.12 10^4^	3.75 10^5^ ± 1.16 10^5^	4.98 10^5^ ± 9.28 10^4^
5'-methylthioinosine	1.07 10^4^ ± 4.81 10^3^	7.71 10^3^ ± 1.76 10^3^	-	-
5'-n-propylthioadenosine	6.31 10^3^ ± 1.78 10^3^	7.59 10^3^ ± 3.32 10^3^	1.55 10^3^ ± 3.38 10^3^	714.56 ± 667.83
9-(beta-D-Ribofuranosyl)zeatin	-	-	9.25 10^3^ ± 6.97 10^3^	1.55 10^3^ ± 2.17 10^3^
Abacavir**	2.94 10^5^ ± 5.29 10^4^	4.70 10^4^ ± 1.97 10^4^	-	1.20 10^6^ ± 2.47 10^5^
Adenosine	1.59 10^4^ ± 3.06 10^3^	7.69 10^3^ ± 2.67 10^3^	3.94 10^4^ ± 7.27 10^3^	1.08 10^4^ ± 1.80 10^3^
adenosine 5'-propyl phosphate	2.51 10^4^ ± 5.19 10^3^	1.42 10^4^ ± 3.88 10^3^	-	1.58 10^4^ ± 4.07 10^3^
Adenosine thiamine diphosphate*	0.02 ± 0.04	-	0.04 ± 0.09	0.11 ± 0.15
Biotinyl-5’-AMP	-	-	-	4.63 10^3^ ± 4.40 10^3^
Cordycepin	6.19 10^5^ ± 7.59 10^4^	2.90 10^3^ ± 6.48 10^3^	3.94 10^6^ ± 1.24 10^6^	1.06 10^6^ ± 1.17 10^5^
Cytidine	1.05 10^4^ ± 2.72 10^3^	4.98 10^3^ ± 4.35 10^3^	1.17 10^3^ ± 2.53 10^3^	4.71 10^4^ ± 1.35 10^4^
Deoxyinosine*	1.86 ± 0.49	-	4.73 ± 3.27	-
Deoxyuridine	1.89 10^4^ ± 3.37 10^3^	481.39 ± 619.93	1.29 10^5^ ± 2.44 10^4^	3.07 10^4^ ± 8.34 10^3^
Entecavir**	7.76 10^3^ ± 4.76 10^3^	9.02 10^3^ ± 5.65 10^3^	3.91 10^4^ ± 1.40 10^4^	-
Guanosine	1.08 10^4^ ± 7.71 10^3^	1.71 10^4^ ± 5.40 10^3^	7.45 10^5^ ± 1.47 10^5^	1.21 10^5^ ± 1.36 10^4^
Imidazoleacetic acid riboside	1.38 10^4^ ± 2.15 10^3^	1.07 10^4^ ± 2.08 10^3^	3.02 10^4^ ± 6.77 10^3^	1.19 10^4^ ± 3.33 10^3^
Inosine	1.91 10^3^ ± 2.95 10^3^	1.72 10^4^ ± 3.90 10^3^	1.38 10^5^ ± 2.62 10^4^	5.95 10^3^ ± 3.36 10^3^
Mizoribine	1.09 10^4^ ± 2.91 10^3^	2.48 10^4^ ± 2.02 10^3^	28.76 ± 64.30	-
N2,N2-Dimethylguanosine	9.43 10^4^ ± 5.49 10^3^	5.89 10^4^ ± 1.07 10^4^	3.02 10^4^ ± 5.38 10^3^	8.05 10^4^ ± 8.22 10^3^
N4-Acetylcytidine*	0.18 ± 0.39	2.44 ± 0.35	-	-
N6,N6-Dimethyladenosine	3.45 10^4^ ± 2.87 10^3^	1.13 10^4^ ± 1.03 10^4^	1.82 10^4^ ± 3.99 10^3^	3.19 10^4^ ± 6.20 10^3^
N6-hydroxyethyl-adenosine	4.25 10^6^ ± 3.09 10^5^	-	1.28 10^6^ ± 4.17 10^5^	5.35 10^6^ ± 4.95 10^5^
N6-methyladenosine	9.03 10^3^ ± 1.28 10^3^	8.91 10^3^ ± 2.99 10^3^	1.31 10^4^ ± 3.51 10^3^	6.63 10^3^ ± 4.16 10^3^
Orotidine	6.76 10^3^ ± 4.19 10^3^	3.14 10^4^ ± 5.21 10^3^	-	-
Pseudouridine	1.12 10^4^ ± 2.70 10^3^	2.56 10^3^ ± 2.56 10^3^	1.10 10^5^ ± 2.08 10^4^	6.78 10^4^ ± 1.08 10^4^
Pyrimidine 5’-deoxynucleotide	1.17 10^4^ ± 3.16 10^3^	5.18 10^3^ ± 2.45 10^3^	4.34 10^3^ ± 2.29 10^3^	4.79 10^4^ ± 5.19 10^3^
Pyrimidine 5’-nucleotide	-	-	3.31 10^3^ ± 5.14 10^3^	-
Pyrimidine nucleoside*	0.93 ± 1.14	-	-	8.35 ± 4.64
Succinyladenosine	4.67 10^4^ ± 1.22 10^4^	4.38 10^4^ ± 1.09 10^4^	2.06 10^4^ ± 1.35 10^4^	2.53 10^4^ ± 1.18 10^4^
Telbivudine**	3.40 10^4^ ± 1.22 10^4^	-	189.15 ± 422.95	8.48 10^4^ ± 8.83 10^3^
Thymidine	822.96 ± 1.54 10^3^	3.28 10^4^ ± 9.15 10^3^	4.31 10^4^ ± 1.91 10^4^	6.02 10^3^ ± 4.48 10^3^
Uridine	1.32 10^4^ ± 8.90 10^3^	7.60 10^3^ ± 6.59 10^3^	-	-
***Water fractions***				
(S)(+)-Allantoin	-	3.52 10^3^ ± 1.97 10^3^	8.19 10^3^ ± 1.77 10^3^	1.09 10^3^ ± 1.01 10^3^
(S)-5'-Deoxy-5'-(methylsulfinyl)adenosine	-	1.25 10^5^ ± 2.49 10^4^	2.95 10^5^ ± 3.33 10^4^	1.76 10^4^ ± 1.87 10^3^
1-Methylguanosine*	1.71 ± 0.71	5.31 ± 0.64	17.18 ± 1.46	11.92 ± 1.11
1-Methylinosine	147.39 ± 328.56	1.28 10^4^ ± 2.97 10^3^	551.89 ± 793.04	227.43 ± 508.54
2-Aminoadenosine*	0.30 ± 0.46	15.55 ± 4.27	0.05 ± 0.11	26.17 ± 1.76
2-Methylaminoadenosine	28.64 ± 48.20	6.33 10^3^ ± 1.97 10^3^	689.02 ± 340.43	1.97 10^4^ ± 1.11 10^3^
3’-amino-3’-deoxyadenosine*	27.21± 8.18	33.26 ± 9.76	39.61 ± 7.65	20.96 ± 7.14
3’-homocitrullyl-amino-3’deoxyadenosine	-	9.63 10^3^ ± 1.54 10^3^	1.49 10^3^ ± 958.95	1.36 10^4^ ± 6.63 10^3^
3-Methyluridine	-	7.86 10^3^ ± 4.71 10^3^	2.95 10^3^ ± 561.69	2.40 10^3^ ± 1.50 10^3^
5’-(aminoiminomethyl)amino-5’-deoxyadenosine dihydrogen sulfate*	-	-	0.55 ± 0.85	21.43 ± 2.94
5-Carboxy-2’-deoxyuridine	-	1.35 10^3^ ± 774.67	1.96 10^3^ ± 256.22	3.86 10^3^ ± 2.19 10^3^
5’-Dehydroadenosine*	1.46 ± 0.56	3.21 ± 0.72	4.73 ± 0.72	17.24 ± 0.95
5-Methyldeoxycytidine	-	1.76 10^4^ ± 5.80 10^3^	1.76 10^4^ ± 6.14 10^3^	7.46 10^4^ ± 1.68 10^4^
5'-Methylthioadenosine	-	1.09 10^5^ ± 2.06 10^4^	1.23 10^6^ ± 2.09 10^5^	1.64 10^5^ ± 1.78 10^4^
5'-methylthioinosine	-	7.39 10^3^ ± 4.82 10^3^	-	335.25 ± 504.88
5'-n-propylthioadenosine	177.62 ± 397.17	7.13 10^3^ ± 1.58 10^3^	1.36 10^3^ ± 112.80	883.12 ± 387.78
Abacavir**	-	1.89 10^5^ ± 6.08 10^4^	1.84 10^5^ ± 3.82 10^4^	2.65 10^6^ ± 4.60 10^5^
Adenosine	-	1.04 10^4^ ± 5.87 10^3^	9.32 10^3^ ± 882.60	1.04 10^4^ ± 5.99 10^3^
Adenosine 5'-propyl phosphate	-	6.42 10^3^ ± 683.61	339.62 ± 244.82	3.86 10^3^ ± 697.66
Adenosine thiamine diphosphate*	-	0.02 ± 0.06	0.01 ± 0.01	0.06 ± 0.08
Biotinyl-5’-AMP	-	2.38 10^3^ ± 1.49 10^3^	966.31 ± 203.20	291.15 ± 651.02
Cordycepin	-	1.78 10^6^ ± 3.17 10^5^	1.13 10^6^ ± 2.27 10^5^	5.91 10^6^ ± 5.10 10^5^
Cytidine	-	-	9.81 10^3^ ± 1.62 10^3^	1.60 10^4^ ± 3.70 10^3^
Deoxyinosine*	0.22 ± 0.16	0.57 ± 0.41	8.39 ± 0.83	-
Deoxyuridine	41.03 ± 91.75	3.39 10^4^ ± 1.97 10^4^	4.35 10^4^ ± 5.86 10^3^	1.19 10^5^ ± 6.81 10^4^
Entecavir**	-	1.19 10^4^ ± 3.58 10^3^	1.43 10^4^ ± 2.14 10^3^	1.31 10^3^ ± 569.50
Guanosine	41.71 ± 75.66	2.02 10^5^ ± 3.68 10^4^	3.34 10^4^ ± 3.60 10^3^	2.85 10^5^ ± 2.84 10^4^
Imidazoleacetic acid riboside	-	3.36 10^3^ ± 1.54 10^3^	2.09 10^4^ ± 2.37 10^3^	1.57 10^4^ ± 9.29 10^3^
Inosine	57.14 ± 127.76	1.17 10^3^ ± 555.66	1.49 10^4^ ± 1.67 10^3^	131.68 ± 240.68
Mizoribine	-	-	1.18 10^4^ ± 2.21 10^3^	-
N2,N2-Dimethylguanosine	-	2.75 10^4^ ± 5.52 10^3^	2.60 10^4^ ± 3.81 10^3^	1.19 10^5^ ± 1.37 10^4^
N4-Acetylcytidine*	0.01 ± 0.03	7.86 ± 1.23	0.05 ± 0.12	0.27 ± 0.22
N6,N6-Dimethyladenosine	1.12 10^3^ ± 646.62	3.28 10^3^ ± 2.99 10^3^	1.26 10^4^ ± 2.39 10^3^	2.98 10^4^ ± 4.30 10^3^
N6-hydroxyethyl-adenosine	-	9.45 10^4^ ± 2.81 10^4^	2.45 10^6^ ± 3.79 10^5^	4.93 10^6^ ± 2.17 10^5^
N6-methyladenosine	414.74 ± 389.21	3.00 10^3^ ± 1.98 10^3^	4.29 10^3^ ± 1.17 10^3^	1.48 10^3^ ± 596.88
Orotidine	-	1.20 10^4^ ± 7.75 10^3^	390.03 ± 358.15	447.55 ± 429.97
Pseudouridine	35.24 ± 78.79	2.94 10^4^ ± 5.50 10^3^	4.51 10^4^ ± 6.83 10^3^	2.19 10^5^ ± 3.11 10^4^
Pyrimidine 5'-deoxynucleotide	-	2.85 10^3^ ± 1.61 10^3^	2.92 10^3^ ± 453.81	5.72 10^3^ ± 3.24 10^3^
Pyrimidine 5'-nucleotide	-	888.66 ± 542.83	1.46 10^3^ ± 244.87	4.81 10^3^ ± 2.87 10^3^
Pyrimidine nucleoside*	-	1.01 ± 0.99	0.06 ± 0.13	12.49 ± 1.45
Succinyladenosine	327.83 ± 449.31	3.27 10^4^ ± 7.38 10^3^	1.33 10^4^ ± 4.38 10^3^	2.72 10^4^ ± 4.78 10^3^
Telbivudine**	-	564.77 ± 657.24	2.22 10^3^ ± 741.22	1.29 10^5^ ± 2.29 10^4^
Thymidine	53.04 ± 118.59	1.11 10^4^ ± 2.42 10^3^	2.82 10^4^ ± 1.63 10^3^	2.31 10^4^ ± 2.37 10^3^
Uridine	-	1.04 10^4^ ± 3.27 10^3^	34.21 ± 76.50	1.47 10^3^ ± 328.24
***n-Hexane fractions***				
(S)-5’-Deoxy-5’-(methylsulfinyl)adenosine*	0.02 ± 0.03	-	-	-
2-Methylaminoadenosine	488.94 ± 490.95	2.99 10^3^ ± 1.59 10^3^	-	327.66 ± 732.67
3’-amino-3’-deoxyadenosine*	0.02 ± 0.04	0.68 ± 0.98	0.21 ± 0.40	1.67 ± 2.48
3-Methyluridine	-	-	1.70 10^3^ ± 2.35 10^3^	-
5’-Dehydroadenosine	-	0.01 ± 0.01	-	-
5-Methyldeoxycytidine	-	-	-	248.96 ± 556.69
5’-n-propylthioadenosine	369.12 ± 825.37	83.22 ± 186.08	-	156.89 ± 350.81
Abacavir**	-	-	134.18 ± 300.04	-
Adenosine	1.12 10^3^ ± 2.51 10^3^	928.00 ± 2.09 10^3^	922.56 ± 2.06 10^3^	-
adenosine 5’-propyl phosphate	-	19.23 ± 42.99	484.06 ± 1.08 10^3^	43.19 ± 96.57
Adenosine thiamine diphosphate*	0.04 ± 0.09	-	0.04 ± 0.08	0.07 ± 0.09
Cytidine	-	177.36 ± 396.59	1.68 10^3^ ± 1.76 10^3^	279.70 ± 625.43
Guanosine	-	-	-	711.88 ± 1.59 10^3^
Imidazoleacetic acid riboside	806.66 ± 1.80 10^3^	951.52 ± 2.13 10^3^	-	-
N2,N2-Dimethylguanosine	1.38 10^3^ ± 3.09 10^3^	-	-	3.16 10^3^ ± 4.38 10^3^
N6,N6-Dimethyladenosine	4.57 10^3^ ± 4.86 10^3^	5.30 10^3^ ± 4.89 10^3^	6.12 10^3^ ± 3.74 10^3^	6.86 10^3^ ± 1.26 10^3^
N6-methyladenosine	694.29 ± 1.55 10^3^	1.99 10^3^ ± 1.93 10^3^	599.58 ± 1.34 10^3^	1.34 10^3^ ± 1.84 10^3^
Pseudouridine	-	-	267.60 ± 498.26	-
Succinyladenosine	2.67 10^3^ ± 2.87 10^3^	1.40 10^3^ ± 1.69 10^3^	654.59 ± 935.31	1.89 10^3^ ± 1.81 10^3^

*Nucleosides detected after dilution steps.

**Nucleoside analogue currently used as pharmaceuticals.

These five markers were generally predominant in methanol fractions, especially the mass intensity of inosine which was always higher independently of the culture medium treatment in these fractions (Fig 6D). Though matrix effect is also strong for water fractions, the mass intensity of adenosine, cordycepin, guanosine, and uridine from SDAY-SS treatments were higher than in methanol ones (Fig 6A–6C and 6E).

Mass intensity values of nucleosides used as markers of quality control of *Cordyceps* products were thoroughly studied (Fig 6). Interestingly, mass intensity of cordycepin extracted in BR was higher in methanol extracts than in water extracts, while the opposite was observed on TA medium (Fig 6A). In the case of adenosine, inosine, and guanosine, higher concentrations were found in the methanol extracts obtained from mycelia grown on BR than on the other culture media tested. Mass intensity values of inosine were two and four times higher with respect to guanosine and adenosine, respectively (Fig 6B and 6C). Uridine was detected in higher concentrations in extracts obtained from synthetic culture media (SDAY and SDAY-SS). Nevertheless, the mass intensity values of uridine compared with the other four markers were too low to consider it as a quality marker for this species (Fig 6E).

### *Cordyceps nidus* is a constitutive source of potential drugs

The metabolome of *C*. *nidus* presented more than 1,000 compounds for each solvent used for extraction. Several of these metabolites corresponded to peptides, vitamins, organic acids and sugars (S3 Table), among which some were recognized some of them as potential bioactive compounds. In Table 3, we depicted the functionality of some metabolites present in methanol fractions of *C*. *nidus*. The metabolites that were selected, were those that were extracted independently of the substrate used for the fungal growth and that were not commonly observed or not previously reported in *Cordyceps*. We also noted that their mass intensities on SDAY were the smaller ones, which is in concordance with the global diversity of metabolites extracted. The mass intensities observed for the other substrate treatments showed to be similar among them.

**Table 3:** Bioactive constitutive metabolites extracted from *C*. *nidus* with methanol. The entries in the table are the means and standard deviations of the mass intensities.

METABOLITES	Reported function	Ref.	SDAY	SDAY-SS	BR	TA
L-Acetylcarnitine	**Acetylating activity**. Activity modulator of nerve growth factor, choline acetyltransferase, and neurotransmitter systems. Antidepressant property.	[43] [44]	65.33 10^2^ ± 14.59 10^2^	63.05 10^5^ ± 15.20 10^5^	22.49 10^6^ ± 16.98 10^5^	33.92 10^5^ ± 54.79 10^4^
2,3-Dehydrosilychristin	Antioxidant activity	[45]	94.19 ± 14.68 10^1^	52.84 10^2^ ± 61.54 10^3^	15.68 10^5^ ± 10.79 10^4^	21.45 10^5^ ± 10.22 10^4^
Phytosphingosine	Antimicrobial, anti-inflammatory, and anti-cancer activity.	[46] [47] [48]	13.81 ± 30.88	14.19 10^5^ ± 28.08 10^4^	12.50 10^5^ ± 32.10 10^4^	11.23 10^5^ ± 15.47 10^4^
Aminocaproic acid	Antifibrinolytic activity.	[49] [50] [51] [52]	10.55 10^2^ ± 14.52 10^2^	97.61 10^4^ ± 16.36 10^4^	72.55 10^4^ ± 64.35 10^3^	15.24 10^5^ ± 58.10 10^3^
Cordysinin B	Antiviral activity.	[53]	75.11 10^1^ ± 11.61 10^2^	28.75 10^4^ ± 48.86 10^3^	80.45 10^3^ ± 94.56 10^2^	92.40 10^4^ ± 14.43 10^4^

### Methanol fractions showed antibiotic activity against gram-positive bacteria

The MIC for each group of samples is listed in Table 4. This activity was found in the genera *Bacillus*, *Enterococcus*, *Kocuria*, and *Staphylococcus*. *Enterococcus faecalis* and *B*. *circulans* showed the largest inhibition halos (with a MIC of 12.5 mg of fungus per 1 mL of solvent) in comparison to the gentamicin control. Satellite bacterial colonies were observed inside the inhibition halos from antimicrobial assays performed with *B*. *circulans*. No antimicrobial activity was present for the water or n-hexane fractions and neither of the extract fractions studied was able to inhibit growth of molds, yeasts, or gram-negative bacteria.

**Table 4:** Minimal Inhibitory Concentration (MIC) of methanol fractions against gram-positive bacteria.

Strain	Extract	MIC (mg mL^-1^)	Diameter of Inhibition zone (cm)	Diameter of inhibition zone for gentamicin control (cm)
*Bacillus cereus*				
	SDAY	25.0	0.60 (± 0.10)	1.64 (± 0.13)
	SDAY-SS	12.5	0.60 (± 0.11)	
	BR	-	-	
	AT	25.0	0.55 (± 0.09)	
*Bacillus circulans*				
	SDAY	12.5*	1.20 (± 0.04)	1.46 (± 0.05)
	SDAY-SS	12.5*	0.85 (± 0.03)	
	BR	12.5*	0.70 (± 0.03)	
	AT	12.5*	1.00 (± 0.01)	
*Enterococcus faecalis*				
	SDAY	12.5	1.20 (± 0.08)	1.00 (± 0.10)
	SDAY-SS	12.5	1.45 (± 0.12)	
	BR	12.5		
	AT	12.5	0.95 (± 0.10)	
*Kocuria rosea*				
	SDAY	50.0	0.60 (± 0.14)	2.10 (± 0.16)
	SDAY-SS	50.0	0.75 (± 0.10)	
	BR	25.0	0.60 (± 0.11)	
	AT	50.0	1.00 (± 0.10)	
*Lysinibacillus sphaericus*				
	SDAY	-	-	1.82 (± 0.10)
	SDAY-SS	50.0	0.75 (± 0.09)	
	BR	-	-	
	AT	12.5	0.60 (± 0.10)	
*Staphylococcus aureus*				
	SDAY	-	-	1.70 (± 0.14)
	SDAY-SS	50.0	0.60 (± 0.09)	
	BR	-	-	
	AT	50.0	0.70 (± 0.11)	
*Staphylococcus epidermidis*				
	SDAY	-	-	2.02 (± 0.05)
	SDAY-SS	50.0	0.75 (± 0.04)	
	BR	-	-	
	AT	25.0	0.70 (0.08)	
*Staphylococcus haemolyticus*				
	SDAY	-	-	2.35 (± 0.04)
	SDAY-SS	25.0	0.75 (± 0.10)	
	BR	50.0	0.60 (± 0.06)	
	AT	25.0	0.65 (± 0.11)	

### *Cordyceps nidus* progressively increased the total phenolic content through time

**Fig 7:**
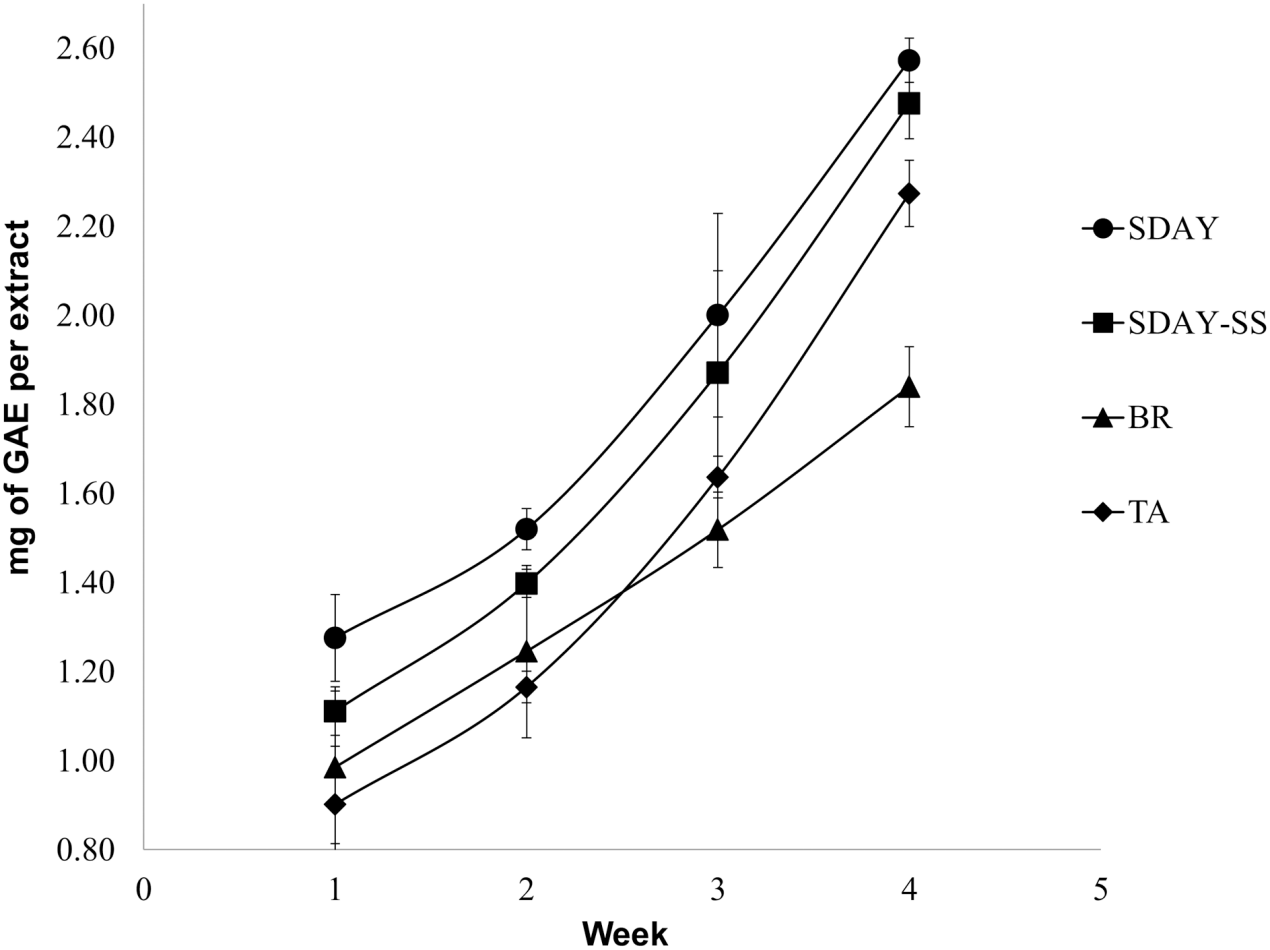
Total phenolic content (TPC) from *C*. *nidus* on different culture media. Error bars correspond to the standard deviation obtained from eight independent replicates.

The TPC of *C*. *nidus* samples grown on different culture media through time are shown in (Fig 7). An exponential increase of TPC is shown for each group of samples on SDAY, SDAY-SS, and TA through time (R^2^ > 0.99). On the other hand, a linear increase of TPC was observed for samples grown on BR medium through time (R^2^ > 0.99). Fungi grown on SDAY for four weeks showed the highest TPC levels in comparison with the other treatments (t-test, p-value<0.05). This phenolic content is followed by that of fungi grown on SDAY-SS and TA medium, respectively. Brown Rice treatment resulted in the lowest TPC levels at the end of the evaluated period. These results are in agreement with the pigmentation patterns observed in the colonies.

## Discussion

In this study, the metabolic profile and pharmacological potential of a species of *Cordyceps* which parasitizes trapdoor spiders were characterized for the first time. At the same time, *Cordyceps nidus* was proposed as a new species closely related to *Cordyceps caloceroides*, a recognized tarantula pathogen. Both species were phylogenetically located in the same subclade of *Cordyceps pruinosa*, a species studied for its potential pharmacological properties, and were closer to the medicinale important fungus *Cordyceps militaris*. Furthermore, the metabolome of *C*. *nidus* has a very diverse composition which is dependent on the substrate where the fungus is grown. The content of nucleosides and particular metabolites found in *C*. *nidus*, especially when it is cultured on semi-natural culture media, suggests its potential for pharmaceutical applications. Another possible application relies on the antimicrobial properties of its metabolites. Methanol fractions presented high activity against gram-positive bacteria, especially *Enterococcus faecalis*, which is known to be highly resistant to antibiotics [54]. We also found the presence of polyphenols in fungal extracts, showing the potential use of *C*. *nidus* as an antioxidant product.

This is the first systematic molecular analysis focusing on the *Cordyceps* pathogens of large spiders belonging to the Mygalomorpha subclass. Additionally, we clarified the phylogenetic position of *Ophiocordyceps caloceroides*, which was firstly placed in the genus *Ophiocordyceps* by Petch in 1933, based on the morphology of its ascospores [42]. Sung et al in 2007, who did not include *O*. *caloceroides* in their molecular analysis, adopted the same result as well [30]. However, our specimens exhibited the same morphology of the type material described by Mains, and the phylogenetic analysis placed them as members of the Cordycipitaceae family [55]. Therefore, we returned this taxon to its original position and nomenclature, *Cordyceps caloceroides* Berk. and Curt. 1869.

*Cordyceps nidus* and *C*. *caloceroides* were located in the same clade of *Cordyceps pruinosa*, a group which is easily recognized by its red pruinose claviform stromata (Fig 2A). The species in this clade share two non-molecular characters. First, the stromata and mycelia turn violet when a drop of KOH is added. This biochemical-physiological reaction was firstly reported by Petch in 1924, when he described *C*. *pruinosa* [56]. Also this reaction was reported for *Metarhizium martialis* [23]. Second, they are associated with hosts that are enclosed in a structure or in a habitat with low oxygen concentration: *Cordyceps ninchukispora* is found in seeds of Lauraceae plants, *C*. *pruinosa* in cocoons of Lepidoptera, *Cordyceps* sp. TL 11464 in oothecae of Orthoptera, *C*. *nidus* in trapdoor spider nests, and *C*. *caloceroides* in deep burrows of Theraphosidae spiders. This suggests that species from KOH+ clade are a differentiated group, phylogenetically isolated by the host showing common environmental condition, i.e., low oxygen concentration. A similar analysis was carried out before recognizing that the species within the “ravanelli” clade in the Ophiocordycipitaceae family, i.e., *Ophiocordyceps heteropoda*, *Ophiocordyceps gracillioides*, *Ophiocordyceps gracillis*, *Ophiocordyceps blattarioides*, and *Ophiocordyceps amazonica*, are recognized as phylogenetic species due to their phylogenetic positions and by their host affiliation and stage of the host, rather than by the slight morphological differences among them [18]. Once again this confirms that the morphological characters in *Cordyceps* s.l. can be acquired or lost through evolution, but the association with a particular host is a factor strongly correlated with their phylogenetic position.

The intracellular composition of *C*. *nidus* is dependent on the substrate used for its culture. The PCAs based on the culture medium used for fungal growth explained more than 50% of the variation in the metabolite composition in polar fractions, especially those extracted with methanol. Among the culture media assayed, BR replicates exhibited the most distinct group of samples. It is known that the culture medium can induce many changes in the physiology of a fungus, and these results are in agreement with other studies involving *Cordyceps* fungi [12]. Environmental characteristics, such as culture media, pH, and temperature, can be determining factors in the production of metabolites [57–59]. Further investigation is warranted re the effect of pH, temperature, and humidity on the production of secondary metabolites, particularly nucleosides, in cultures of *C*. *nidus*. Our results showed that *C*. *nidus* produced a high number of nucleosides, especially in natural culture media, showing its potential use as a therapeutic agent due to the different biological activities reported for these compounds [11]

In this study, we focused our analyses on four nucleosides that are used as markers of quality control in *Cordyceps* s.l.: cordycepin, adenosine, inosine, and guanosine. The production of uridine was not significant in any of the four media assessed. As expected, n-hexane was not effective for nucleosides extraction due to its high polarity. Natural culture media can be used for growing *C*. *nidus* in future studies and for inducing the production of nucleosides. Brown Rice medium was particularly interesting due to the high fungus biomass production on it. Curiously, we found that the presence of sodium selenite did not induce the production of adenosine and cordycepin, which is in disagreement with the promoting activity of selenium on nucleosides produced by *C*. *militaris* [15]. Selenium complexes can promote the activity of several enzymes involved in cordycepin synthesis [60]. Nevertheless, sodium selenite composition was adjusted based on this previous work. Thus, an experimental design using higher compositions may increase the nucleoside production.

Interestingly, some compounds present in *C*. *nidus* are related to active compounds involved in neuronal systems, such as L-acetylcarnitine [43–44], aminocaproic acid [49–52], pregabalin [61], amobarbital [62], and rivastigmine [63]. Among these, L-Acetylcarnitine was predominant in *C*. *nidus* mycelia, reaching mass intensity values up to 10^7^. Furthermore, this compound was not substrate-dependent. Some *Cordyceps* spp. were characterized as agents that prevent neuronal cell death and protective neurons agents [64–66]. These findings suggest that *C*. *nidus* would be a source of compounds for neuronal disease treatments. On the other hand, we also found several metabolites associated as antimicrobials and antioxidants, which is in agreement with the tests performed in this study. Remarkably, in this field metabolites such as cordysinin B are associated with antivirals, which is promising for further studies [53]. Finally, three nucleoside analogues (Abacavir, Entecavir, and Telbivudine) are important to mention. Abacavir and Telbivudine are used to prevent and treat hepatitis B infection [67–68], while Entecavir is a prevention and treatement medication for HIV/AIDS disease [69]. These three metabolites are currently produced by synthetic chemical processes, making *C*. *nidus* a promising natural source of these antiviral compounds.

Strong bactericide activity against highly resistant bacteria, such as *E*. *faecalis*, *Bacillus* spp., and *Staphylococcus* sp., was found in methanol fractions of *C*. *nidus*. These results suggest a novel application against bacteria with high resistance to conventional antibiotics [70]. *Enterococcus* is a genus of gram-positive bacteria and some of its species are opportunistic pathogens. However, these bacteria are more important because they are highly resistant to conventional antibiotics and present high rates of horizontal gene transfer [71]. Therefore, finding alternatives to control these bacteria could be of great utility in medicine. On the other hand, the genus *Bacillus* is one of the most predominant in the environment. *Bacillus* spp. are able to produce endospores and have shown to be resistant to antibiotics [72]. Thus, new alternative compounds for disease treatments are needed. An important observation was the retarded growth of *B*. *circulans* indicated by the inhibition halos, or the presence of small colonies, associated with persistence of the bacteria or bacteriostatic activity of the extracts [73].

In the metabolite profile of the methanol fractions of *C*. *nidus*, several chemical components were found which could potentially act as bactericide. Chloramphenicol, sphingolipids, azoles, penicillin, and other lactams are some of these compounds present in *C*. *nidus* extracts that are well-documented to have antimicrobial activity [74–77]. Nevertheless, imidazole-4-acetaldehyde was only present in the methanol fraction, and its concentration was highest when *C*. *nidus* was grown in SDAY and AT media. Given that we found more activity in extracts derived from these treatments, we suggest that this azole could be a candidate for biological activity against gram-positive bacteria. On the other hand, the presence of ophiocordin did not have an effect on tested fungi possibly due to its low concentration in the extracts. Thus, for future studies, we suggest the performance of antifungal sensitivity tests using concentrations higher than 50 mg per mL of solvent.

Polyphenols are important constituents of fungi, given that they can act as antioxidants due to their capacity to scavenge free radicals [78]. The TPCs of *C*. *nidus* cultivated under various substrates indicate that synthetic culture media are perhaps the best substrates to produce antioxidants. To further assess the antioxidant properties of *C*. *nidus* extracts, antiradical analysis with 2, 2-azinobis (3-ethylbenzothiazoline-6-sulfonic acid) (ABTS) or 2, 2-diphenyl-1-picrylhydrazyl (DPPH) should be performed in the future. Nevertheless, the levels of TPCs on all treatments are still low in comparison with those obtained from cultures of *C*. *pruinosa*, the closer phylogenetically related species [8]. The optimization of other environmental factors is needed to induce the production of higher concentrations of this kind of compounds. The increase of phenols through time is also a remarkable characteristic of this test. A previous analysis established that the concentration of antioxidants is strongly related to the production of pigments by the fungus, thus explaining the increase of TPCs through time [79]. Furthermore, better results for antioxidant production have been obtained by inducing the formation of fruiting bodies [7–8, 13].

## Conclusions

This is the first study that attempts to address the systematics of *Cordyceps* which parasitize Mygalomorpha spiders in the Neotropics, and helps to clarify the taxonomy of specimens in the pictures tagged as *Cordyceps* (*Ophiocordyceps*) *caloceroides* and *Cordyceps nidus* published on the internet (e.g. http://mushroomobserver.org/observer/observation_search?pattern=cordyceps+caloceroides). In the future, a thorough systematic and ecological study will be necessary to reach a more comprehensive understanding of the *Cordyceps*-spiders interaction. This study showed that there are differences in the intracellular metabolite composition of *Cordyceps nidus* when grown on different substrates. We also explored the potential of this newly described pathogen of spiders as a source of biotechnological products. The nucleosides content and the presence of the particular metabolites described above suggested that this *Cordyceps* species may be suitable as a dietary supplement or pharmacological product. Nevertheless, studies of toxicity and edibility are needed to complement the results obtained in this research. We also tested the extracts of *C*. *nidus* in susceptibility antimicrobial tests and we found activity against various important bacteria in clinical environments, such as *Staphylococcus* spp., *Enterococcus* spp., and *Bacillus* spp., among others. From our assays, we found that methanol extractions had the highest activity against these pathogens, especially against *E*. *faecalis*, which is highly resistant to antibiotics. Finally, total phenolic content from these extracts showed the potential of *C*. *nidus* as a source of antioxidant products. Nevertheless, we suggested assessing antiradical activity by stable free radical scavenging analyses with ABTS or DPPH.

## Supporting information

S1 Fig Venn diagram for compounds reported. A) methanol, B) water, and C) n-hexane fractions.
(TIF)

S1 Table GenBank accession numbers for taxa included in this study. (XLSX)

S2 Table Bacterial and fungal strains used in the antimicrobial susceptibility assays. (XLSX)

S3 Table Metabolite composition from cultured *Cordyceps nidus*. (XLSX)

## References

[1] Newman DJ, Cragg GM. Natural products as sources of new drugs over the 30 years from 1981 to 2010. J Nat Prod. 2012;75(3):311–35. doi: 10.1021/np200906s 22316239 10.1021/np200906s PMC3721181

[2] Holliday JC, Cleaver MP. Medicinal value of the caterpillar fungi species of the genus Cordyceps (Fr.) Link (Ascomycetes). A Review. Int J Med Mushrooms. 2008; 10(3):219–34. doi: 10.1615/IntJMedMushr.v10.i3.30

[3] Zhang Y, Zhang C, Li Y, Ma S, Wang C, Xiang M, et al Phylogeography and evolution of a fungal–insect association on the Tibetan Plateau. Mol.Ecol.2014; 23, 5337–5355. doi: 10.1111/mec.12940 25263531 10.1111/mec.12940

[4] Li SP, Yang FQ, Tsim KWK. Quality control of Cordyceps sinensis, a valued traditional Chinese medicine. J Pharm Biomed Anal. 2006; 41(5):1571–84. doi: 10.1016/j.jpba.2006.01.046 16504449 10.1016/j.jpba.2006.01.046

[5] Chioza A, Ohga S, A review on fungal isolates reported as anamorphs of Ophiocordyceps sinensis. J Mycol. 2014; Article ID 913917, 5 pages, 2014. doi: 10.1155/2014/913917

[6] Guo L-X, Xu X-M, Liang F-R, Yuan J-P, Peng J, Wu C-F, et al Morphological observations and fatty acid composition of indoor-cultivated Cordyceps sinensis at a high-altitude laboratory on Sejila Mountain, Tibet. PLoS ONE 2015; 10(5): e0126095 doi: 10.1371/journal.pone.0126095 25938484 10.1371/journal.pone.0126095 PMC4418754

[7] Hyun S-H, Lee S-Y, Sung G-H, Kim SH, Choi H-K. Metabolic profiles and free radical scavenging activity of Cordyceps bassiana fruiting bodies according to developmental stage. PLoS ONE 2013; 8(9): e73065 doi: 10.1371/journal.pone.0073065 24058459 10.1371/journal.pone.0073065 PMC3772819

[8] Oh T-J, Hyun S-H, Lee S-G, Chun Y-J, Sung G-H, Choi H-K. NMR and GC-MS based metabolic profiling and free-radical scavenging activities of Cordyceps pruinosa mycelia cultivated under different media and light conditions. PLoS ONE. 2014; 9(3):e90823 doi: 10.1371/journal.pone.0090823 24608751 10.1371/journal.pone.0090823 PMC3946585

[9] Shrestha B, Zhang W, Zhang Y, Liu X. The medicinal fungus Cordyceps militaris: research and development. Mycol Prog. 2012; 11(3):599–614. doi: 10.1007/s11557-012-0825-y

[10] King AE, Ackley MA, Cass CE, Young JD, Baldwin SA. Nucleoside transporters: from scavengers to novel therapeutic targets. Trends Pharmacol Sci. 2006 8;27(8):416–25. doi: 10.1016/j.tips.2006.06.004 16820221 10.1016/j.tips.2006.06.004

[11] Zhao J, Xie J, Wang LY, Li SP. Advanced development in chemical analysis of Cordyceps. J Pharm Biomed Anal. 2014; 87:271–89. doi: 10.1016/j.jpba.2013.04.025 23688494 10.1016/j.jpba.2013.04.025

[12] Hyun S-H, Lee S-Y, Park SJ, Kim DY, Chun Y-J, Sung G-H, et al Alteration of media composition and light conditions change morphology, metabolic profile, and beauvericin biosynthesis in Cordyceps bassiana mycelium. J Microbiol Biotechnol. 2013; 23(1):47–55. doi: 10.4014/jmb.1208.08058 23314367 10.4014/jmb.1208.08058

[13] Park SJ, Hyun S-H, Suh HW, Lee S-Y, Sung G-H, Kim SH, et al Biochemical characterization of cultivated Cordyceps bassiana mycelia and fruiting bodies by 1H nuclear magnetic resonance spectroscopy. Metabolomics. 2013; 9(1):236–46. doi: 10.1007/s11306-012-0442-4

[14] Dong JZ, Lei C, Zheng XJ, Ai XR, Wang Y, Wang Q. Light Wavelengths Regulate Growth and Active Components of Cordyceps militaris Fruit Bodies. J Food Biochem. 2013; 37(5):578–84. doi: 10.1111/jfbc.12009

[15] Dong JZ, Liu MR, Lei C, Zheng XJ, Wang Y. Effects of selenium and light wavelengths on liquid culture of Cordyceps militaris Link. Appl Biochem Biotechnol. 2012 4;166(8):2030–6. doi: 10.1007/s12010-012-9628-5 22434354 10.1007/s12010-012-9628-5

[16] Yang FQ, Li SP. Effects of sample preparation methods on the quantification of nucleosides in natural and cultured Cordyceps. J Pharm Biomed Anal. 2008 9 10;48(1):231–5. doi: 10.1016/j.jpba.2008.05.012 18573632 10.1016/j.jpba.2008.05.012

[17] Li C, Li Z, Fan M, Cheng W, Long Y, Ding T, et al The composition of Hirsutella sinensis, anamorph of Cordyceps sinensis. J Food Compos Anal. 2006; (8):800–5. doi: 10.1016/j.jfca.2006.04.007

[18] Sanjuan TI, Franco-Molano AE, Kepler RM, Spatafora JW, Tabima J, Vasco-Palacios AM, et al Five new species of entomopathogenic fungi from the Amazon and evolution of neotropical Ophiocordyceps. Fungal Biol. 2015 10;119(10):901–16. doi: 10.1016/j.funbio.2015.06.010 26399185 10.1016/j.funbio.2015.06.010

[19] Stensrud Ø, Hywel-Jones NL, Schumacher T. Towards a phylogenetic classification of Cordyceps: ITS nrDNA sequence data confirm divergent lineages and paraphyly. Mycol Res. 2005; 109(1):41–56. doi: 10.1017/S095375620400139X 15736862 10.1017/s095375620400139x

[20] Fong YK, Anuar S, Lim HP, Tham FY, Sanderson FR. A modified filter paper technique for long-term preservation of some fungal cultures. Mycologist. 2000 8 1;14(3):127–30. doi: 10.1016/S0269-915X(00)80090-7

[21] Kornerup A, Wanscher JH, Pavey D. Methuen handbook of colour. Hastings House; 1984. 260 p.

[22] Küppers H. Atlas de los colores. Madrid: Blume; 1979.

[23] Kepler RM, Sung G-H, Ban S, Nakagiri A, Chen M-J, Huang B, li Z, et al New teleomorph combinations in the entomopathogenic genus Metacordyceps. Mycologia. 2012 2;104(1):182–97. doi: 10.3852/11-070 22067304 10.3852/11-070

[24] Sanjuan T, Tabima J, Restrepo S, Læssøe T, Spatafora JW, Franco-Molano AE. Entomopathogens of Amazonian stick insects and locusts are members of the Beauveria species complex (Cordyceps sensu stricto). Mycologia. 2014; 106(2):260–75. doi: 10.3852/106.2.260 24782494 10.3852/106.2.260

[25] Kearse M, Moir R, Wilson A, Stones-Havas S, Cheung M, Sturrock S, Buxton S, et al Geneious Basic: an integrated and extendable desktop software platform for the organization and analysis of sequence data. Bioinforma Oxf Engl. 2012; 28(12):1647–9. doi: 10.1093/bioinformatics/bts199 22543367 10.1093/bioinformatics/bts199 PMC3371832

[26] Hall T. BioEdit: a user-friendly biological sequence alignment editor and analysis program for Windows 95/98/NT. Nucleic Acids Symp Ser. 1999;41:95–8.

[27] Chiriví-Salomón JS, Danies G, Restrepo S, Sanjuan T. Lecanicillium sabanense sp. nov. (Cordycipitaceae) a new fungal entomopathogen of coccids. Phytotaxa. 2015; 234(1):63–74. doi: 10.11646/phytotaxa.234.1.4

[28] Johnson D, Sung G-H, Hywel-Jones NL, Luangsa-Ard JJ, Bischoff JF, Kepler RM, et al Systematics and evolution of the genus Torrubiella (Hypocreales, Ascomycota). Mycol Res. 2009; 113(3):279–89. doi: 10.1016/j.mycres.2008.09.008 18938242 10.1016/j.mycres.2008.09.008

[29] Rehner SA, Buckley E. A Beauveria phylogeny inferred from nuclear ITS and EF1-alpha sequences: evidence for cryptic diversification and links to Cordyceps teleomorphs. Mycologia. 2005 2;97(1):84–98. 16389960 10.3852/mycologia.97.1.84

[30] Sung G-H, Hywel-Jones NL, Sung J-M, Luangsa-ard JJ, Shrestha B, Spatafora JW. Phylogenetic classification of Cordyceps and the clavicipitaceous fungi. Stud Mycol. 2007;57:5–59. doi: 10.3114/sim.2007.57.01 18490993 10.3114/sim.2007.57.01 PMC2104736

[31] Miller MA, Pfeiffer W, Schwartz T, Creating the CIPRES Science Gateway for inference of large phylogenetic trees. In Proceedings of the Gateway Computing Environments Workshop (GCE), 11 April. 2015, New Orleans, LA pp 1–8

[32] Stamatakis A, RAxML-VI_HPC: maximum likelihood-based phylogenetic analyses with thousands of taxa and mixed models. Bioinforma OXf Engl. 2006; 22(21): 2688–90. doi: 10.1093/bioinformatics/btl446 16928733 10.1093/bioinformatics/btl446

[33] Huelsenbeck JP, Ronquist F, Nielsen R, Bollback JP. Bayesian inference of phylogeny and its impact on evolutionary biology. Science. 2001 12 14;294(5550):2310–4. doi: 10.1126/science.1065889 11743192 10.1126/science.1065889

[34] Drummond AJ, Rambaut A. BEAST: Bayesian evolutionary analysis by sampling trees. BMC Evol Biol. 2007; 7(1):214 doi: 10.1186/1471-2148-7-214 17996036 10.1186/1471-2148-7-214 PMC2247476

[35] Kim S-Y, Shrestha B, Sung G-H, Han S-K, Sung J-M. Optimum Conditions for Artificial Fruiting Body Formation of Cordyceps cardinalis. Mycobiology. 2010; 38(2):133–6. doi: 10.4489/MYCO.2010.38.2.133 23956641 10.4489/MYCO.2010.38.2.133 PMC3741564

[36] Harvey-Clark C. IACUC Challenges in Invertebrate Research. ILAR J. 2011 1 1;52(2):213–20. 21709314 10.1093/ilar.52.2.213

[37] Lisec J, Schauer N, Kopka J, Willmitzer L, Fernie AR. Gas chromatography mass spectrometry-based metabolite profiling in plants. Nat Protoc. 2006;1(1):387–96. doi: 10.1038/nprot.2006.59 17406261 10.1038/nprot.2006.59

[38] Clinical and Laboratory Standards Institute (CLSI). Method for Antifungal Disk Diffusion Susceptibility Testing of Yeasts; Approved Guideline-Second Edition. 2nd ed. 950 West Valley Road, Suite 2500, Wayne, Pennsylvania 19087 USA: Clinical and Laboratory Standards Institute; 2009. (NCCLS document M44-A; vol. 29).

[39] Clinical and Laboratory Standards Institute (CLSI). Method for Antifungal Disk Diffusion Susceptibility Testing of Filamentous Fungi: Approved Guideline. 1st ed. 950 West Valley Road, Suite 2500, Wayne, Pennsylvania 19087 USA: Clinical and Laboratory Standards Institute; 2010. (NCCLS document M51-A; vol. 30).

[40] Clinical and Laboratory Standards Institute (CLSI). Performance Standards for Antimicrobial Disk Susceptibility Tests; Approved Standard-Eleventh Edition. 11th ed. 950 West Valley Road, Suite 2500, Wayne, Pennsylvania 19087 USA: Clinical and Laboratory Standards Institute; 2012. (CLSI document M02-A11; vol. 32).

[41] R Development Core Team. R: a language and environment for statistical computing [Internet]. Vienna, Austria; 2011 [cited 2015 Sep 28]. http://www.R-project.org/

[42] Petch T. Notes on entomogenous fungi. Trans Br Mycol Soc. 1933 8 16;18(1):48–75.

[43] Nasca C, Xenos D, Barone Y, Caruso A, Scaccianoce S, Matrisciano F, et al L-acetylcarnitine causes rapid antidepressant effects through the epigenetic induction of mGlu2 receptors. Proc Natl Acad Sci U S A. 2013 3 19;110(12):4804–9. doi: 10.1073/pnas.1216100110 23382250 10.1073/pnas.1216100110 PMC3607061

[44] Chiechio S, Copani A, Nicoletti F, Gereau R IV. L-Acetylcarnitine: A Proposed Therapeutic Agent for Painful Peripheral Neuropathies. Curr Neuropharmacol. 2006 7;4(3):233–7. doi: 10.2174/157015906778019509 18615142 10.2174/157015906778019509 PMC2430690

[45] Tong S, Chu C, Wei Y, Wang L, Gao X, Xu X, et al Preparation and effects of 2,3-dehydrosilymarin, a promising and potent antioxidant and free radical scavenger. J Pharm Pharmacol. 2011 2;63(2):238–44. doi: 10.1111/j.2042-7158.2010.01210.x 21235588 10.1111/j.2042-7158.2010.01210.x

[46] Park M-T, Kim M-J, Kang Y-H, Choi S-Y, Lee J-H, Choi J-A, et al Phytosphingosine in combination with ionizing radiation enhances apoptotic cell death in radiation-resistant cancer cells through ROS-dependent and -independent AIF release. Blood. 2005 2 15;105 (4):1724–33. doi: 10.1182/blood-2004-07-2938 15486061 10.1182/blood-2004-07-2938

[47] Pavicic T, Wollenweber U, Farwick M, Korting HC. Anti-microbial and -inflammatory activity and efficacy of phytosphingosine: an in vitro and in vivo study addressing acne vulgaris. Int J Cosmet Sci. 2007 6;29(3):181–90. doi: 10.1111/j.1467-2494.2007.00378.x 18489348 10.1111/j.1467-2494.2007.00378.x

[48] Fischer CL, Drake DR, Dawson DV, Blanchette DR, Brogden KA, Wertz PW. Antibacterial activity of sphingoid bases and fatty acids against Gram-positive and Gram-negative bacteria. Antimicrob Agents Chemother. 2012 3;56(3):1157–61. doi: 10.1128/AAC.05151-11 22155833 10.1128/AAC.05151-11 PMC3294957

[49] Tzortzopoulou A, Cepeda MS, Schumann R, Carr DB. Antifibrinolytic agents for reducing blood loss in scoliosis surgery in children. Cochrane Database Syst Rev. 2008 7 16;(3):CD006883 doi: 10.1002/14651858.CD006883.pub2 18646174 10.1002/14651858.CD006883.pub2

[50] Golembiewski J. Antifibrinolytic Use in the Perioperative Setting: Aminocaproic Acid and Tranexamic Acid. J Perianesth Nurs. 2015 12 1;30(6):560–3. doi: 10.1016/j.jopan.2015.09.005 26596393 10.1016/j.jopan.2015.09.005

[51] Estcourt LJ, Desborough M, Brunskill SJ, Doree C, Hopewell S, Murphy MF, et al Antifibrinolytics (lysine analogues) for the prevention of bleeding in people with haematological disorders. Cochrane Database Syst Rev. 2016 3 15;3:CD009733 doi: 10.1002/14651858.CD009733.pub3 26978005 10.1002/14651858.CD009733.pub3 PMC4838155

[52] Buckley LF, Reardon DP, Camp PC, Weinhouse GL, Silver DA, Couper GS, et al Aminocaproic acid for the management of bleeding in patients on extracorporeal membrane oxygenation: Four adult case reports and a review of the literature. Heart Lung J Crit Care. 2016 6;45(3):232–6. doi: 10.1016/j.hrtlng.2016.01.011 26907195 10.1016/j.hrtlng.2016.01.011

[53] Yu Y, Jiang Z, Song W, Yang Y, Li Y, Jiang J, et al Glucosylated caffeoylquinic acid derivatives from the flower buds of Lonicera japonica. Acta Pharm Sin B. 2015 5;5(3):210–4. doi: 10.1016/j.apsb.2015.01.012 26579448 10.1016/j.apsb.2015.01.012 PMC4629231

[54] Kristich CJ, Rice LB, Arias CA. Enterococcal Infection—Treatment and Antibiotic Resistance In: Gilmore MS, Clewell DB, Ike Y, Shankar N, editors. Enterococci: From Commensals to Leading Causes of Drug Resistant Infection [Internet]. Boston: Massachusetts Eye and Ear Infirmary; 2014 [cited 2016 Jul 7]. http://www.ncbi.nlm.nih.gov/books/NBK190420/

[55] Mains EB. Species of *Cordyceps* on Spiders. Bull Torrey Bot Club. 1954;81(6):492–500. doi: 10.2307/2481945

[56] Petch T. Xylariaceae Zeylanicae. Ann R Bot Gard Perad. 1924;8:119–66.

[57] VanderMolen KM, Raja HA, El-Elimat T, Oberlies NH. Evaluation of culture media for the production of secondary metabolites in a natural products screening program. AMB Express. 2013 12 17; 3:71 doi: 10.1186/2191-0855-3-71 24342059 10.1186/2191-0855-3-71 PMC3917616

[58] Elias BC, Said S, de Albuquerque S, Pupo MT. The influence of culture conditions on the biosynthesis of secondary metabolites by Penicillium verrucosum Dierck. Microbiol Res. 2006 7 3;161(3):273–80. doi: 10.1016/j.micres.2005.10.003 16765844 10.1016/j.micres.2005.10.003

[59] Zeilinger S, Martín J-F, García-Estrada C. Biosynthesis and Molecular Genetics of Fungal Secondary Metabolites. Springer; 2015 263 p.

[60] Dong JZ, Lei C, Ai XR, Wang Y. Selenium Enrichment on Cordyceps militaris Link and Analysis on Its Main Active Components. Appl Biochem Biotechnol. 2012, 166: 1215–1224. doi: 10.1007/s12010-011-9506-6 22246726 10.1007/s12010-011-9506-6

[61] Taylor CP, Angelotti T, Fauman E. Pharmacology and mechanism of action of pregabalin: the calcium channel alpha2-delta (alpha2-delta) subunit as a target for antiepileptic drug discovery. Epilepsy Res. 2007, 73(2):137–50. doi: 10.1016/j.eplepsyres.2006.09.008 17126531 10.1016/j.eplepsyres.2006.09.008

[62] Peng L, Hertz L. Amobarbital inhibits K+-stimulated glucose oxidation in cerebellar granule neurons by two mechanisms. Eur J Pharmacol. 2002 6 20;446(1–3):53–61. 12098585 10.1016/s0014-2999(02)01794-6

[63] Müller T. Rivastigmine in the treatment of patients with Alzheimer’s disease. Neuropsychiatr Dis Treat. 2007, 3(2):211–8. 19300554 10.2147/nedt.2007.3.2.211 PMC2654625

[64] Jin D-Q, Park B-C, Lee J-S, Choi H-D, Lee Y-S, Yang J-H, et al Mycelial extract of Cordyceps ophioglossoides prevents neuronal cell death and ameliorates beta-amyloid peptide-induced memory deficits in rats. Biol Pharm Bull. 2004, 27(7):1126–9. doi: 10.1248/bpb.27.1126 15256753 10.1248/bpb.27.1126

[65] Hwang IK, Lim SS, Yoo K-Y, Lee YS, Kim HG, Kang I-J, et al A Phytochemically Characterized Extract of Cordyceps militaris and Cordycepin Protect Hippocampal Neurons from Ischemic Injury in Gerbils A. Planta Med. 2008, 74(2):114–9. doi: 10.1055/s-2008-1034277 18214814 10.1055/s-2008-1034277

[66] Liu Z, Li P, Zhao D, Tang H, Guo J. Protective effect of extract of Cordyceps sinensis in middle cerebral artery occlusion-induced focal cerebral ischemia in rats. Behav Brain Funct BBF. 2010; 6:61 doi: 10.1186/1744-9081-6-61 20955613 10.1186/1744-9081-6-61 PMC2984477

[67] Yuen GJ, Weller S, Pakes GE. A review of the pharmacokinetics of abacavir. Clin Pharmacokinet. 2008; 47(6):351–71. doi: 10.2165/00003088-200847060-00001 18479171 10.2165/00003088-200847060-00001

[68] Osborn M. Safety and efficacy of telbivudine for the treatment of chronic hepatitis B [Internet]. Therapeutics and Clinical Risk Management. 2009 [cited 2016 Dec 15]. https://www.dovepress.com/safety-and-efficacy-of-telbivudine-for-the-treatment-of-chronic-hepati-peer-reviewed-article-TCRM 10.2147/tcrm.s5318 PMC2762437 19851526

[69] Sims KA, Woodland AM. Entecavir: a new nucleoside analog for the treatment of chronic hepatitis B infection. Pharmacotherapy. 2006, 26(12):1745–57. doi: 10.1592/phco.26.12.1745 17125436 10.1592/phco.26.12.1745

[70] Grant SS, Hung DT. Persistent bacterial infections, antibiotic tolerance, and the oxidative stress response. Virulence. 2013, 4(4):273–83. doi: 10.4161/viru.23987 23563389 10.4161/viru.23987 PMC3710330

[71] Vu J, Carvalho J. Enterococcus: review of its physiology, pathogenesis, diseases and the challenges it poses for clinical microbiology. Front Biol. 2011, 6(5):357–66. doi: 10.1007/s11515-011-1167-x

[72] Nicholson WL. Roles of Bacillus endospores in the environment. Cell Mol Life Sci CMLS. 2002, 59(3):410–6. doi: 10.1007/s00018-002-8433-7 11964119 10.1007/s00018-002-8433-7 PMC11337551

[73] Nemeth J, Oesch G, Kuster SP. Bacteriostatic versus bactericidal antibiotics for patients with serious bacterial infections: systematic review and meta-analysis. J Antimicrob Chemother. 2015, 70(2):382–95. doi: 10.1093/jac/dku379 25266070 10.1093/jac/dku379

[74] Tarani Prakash S, Umesh Kumar P, Satyendra G, Meghna S. Divers Pharmacological Significance of Imidazole Derivatives-A Review. Res J Pharm Technol. 2013;6:44–50.

[75] Gupta KG, Kumar V, Kaur K. Imidazole Containing Natural Products as Antimicrobial Agents: A Review. Nat Prod J. 2014, 4(2):73–81.

[76] Wisseman CL, Smadel JE, Hahn FE, Hopps HE. Mode of Action of Chloramphenicol I. Action of Chloramphenicol on Assimilation of Ammonia and on Synthesis of Proteins and Nucleic Acids in Escherichia coli. J Bacteriol. 1954, 67(6):662–673. 13174493 10.1128/jb.67.6.662-673.1954 PMC357302

[77] Waxman DJ, Strominger JL. Penicillin.Binding Proteins and the Mecanism of Action of β-lactam antibiotics. Ann. Rev. Biochem. 1983, 52: 825–69. doi: 10.1146/annurev.bi.52.070183.004141 6351730 10.1146/annurev.bi.52.070183.004141

[78] Palacios I, Lozano M, Moro C, D’Arrigo M, Rostagno A, Martínez JA, García-Lafuente A, Guillamón E, Villares A. Antioxidant Properties of Phenolic Compounds Occuring in Edible Mushrooms. Food Chemistry. 2011, 128(3):674–78. doi: 10.1016/j.foodchem.2011.03.085

[79] Shcherba VV, Babitskaya VG, Kurchenko VP, Ikonnikova NV, Kukulyanskaya TA. Antioxidant Properties of Fungal Melanin Pigments. Appl Biochem Microbiol. 2000, 36(5):491–95. doi: 10.1007/BF02731896 11042882

