# Supplementary figures and images for: Metabolomic profile and nucleoside composition of Cordyceps nidus sp. nov. (Cordycipitaceae): A new source of active compounds
Source: PLoS One. 2017 Jun 21;12(6):e0179428. doi: 10.1371/journal.pone.0179428 (PMC5479552; doi:10.1371/journal.pone.0179428)

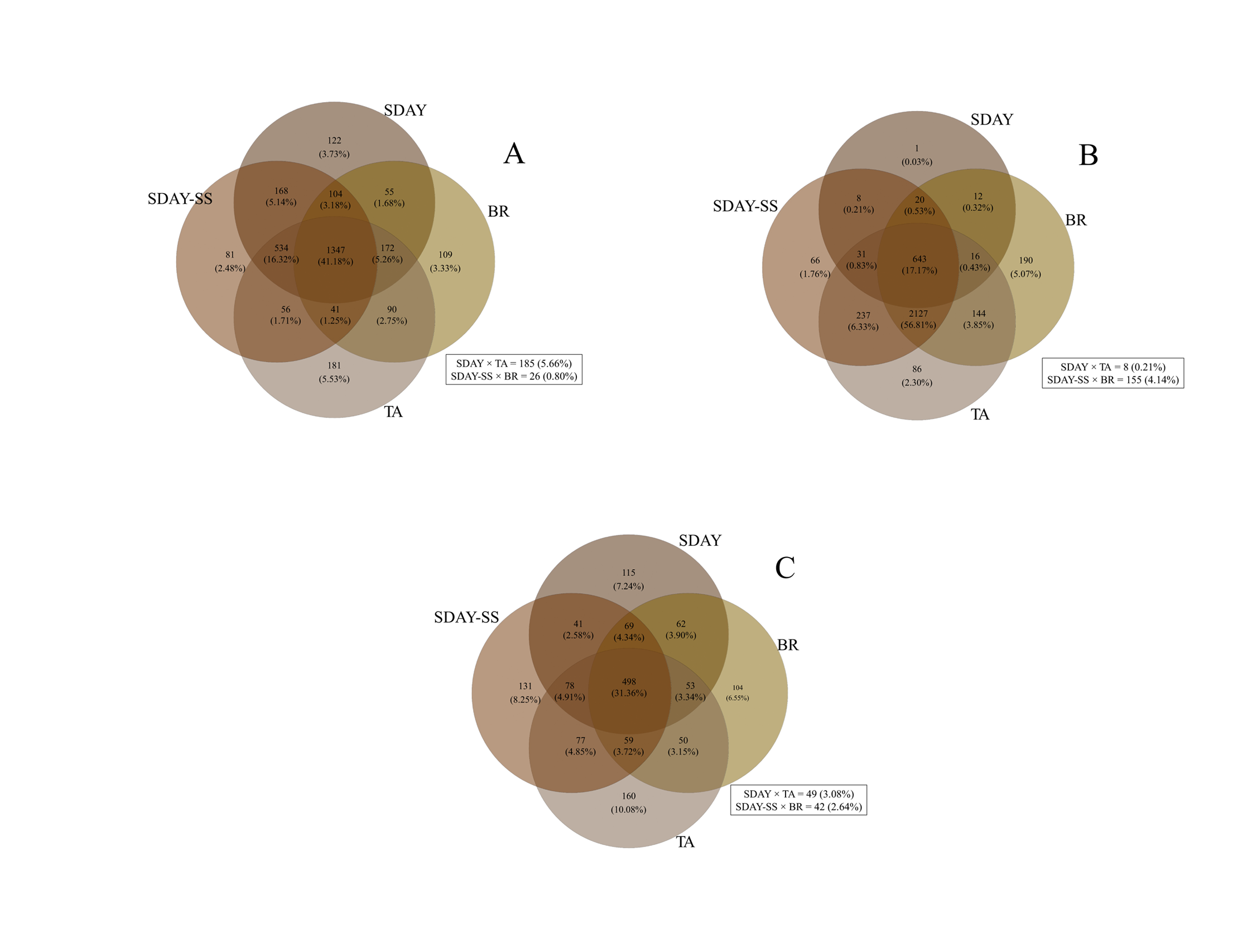

Supplement: S1 Fig — A) methanol, B) water, and C) n-hexane fractions. (TIF) [file pone.0179428.s001.tif]
